# A scoping review of the health effects of fermented foods in specific human populations and their potential role in precision nutrition: current knowledge and gaps

**DOI:** 10.3389/fnut.2025.1650633

**Published:** 2025-11-13

**Authors:** Christèle Humblot, Panagiota Alvanoudi, Emilia Alves, Ricardo Assunçao, Miona Belovic, Tugce Bulmus-Tuccar, Christophe Chassard, Muriel Derrien, Mustafa Fevzi Karagöz, Sibel Karakaya, Marta Laranjo, Fani Th Mantzouridou, Catarina Rosado, Smilja Pracer, Helen Saar, Julien Tap, Primož Treven, Guy Vergères, Eugenia Pertziger, Isabelle Savary-Auzeloux

**Affiliations:** 1 QualiSud, Université de Montpellier, Avignon Université, CIRAD, Institut Agro, IRD, Université de la Réunion, Montpellier, France; 2 French National Research Institute for Sustainable Development (IRD), Montpellier, France; 3 Laboratory of Food Chemistry and Technology, School of Chemistry, Aristotle University of Thessaloniki, Thessaloniki, Greece; 4 CBIOS - Universidade Lusófona's Research Center for Biosciences & Health Technologies, Lisbon, Portugal; 5 Egas Moniz Center for Interdisciplinary Research (CiiEM), Egas Moniz School of Health & Science, Caparica, Portugal; 6 Food and Nutrition Department, National Institute of Health, Dr. Ricardo Jorge, Lisbon, Portugal; 7 Institute of Food Technology in Novi Sad, University of Novi Sad, Novi Sad, Serbia; 8 Department of Nutrition and Dietetics, Yüksek Ihtisas University, Ankara, Türkiye; 9 Université Clermont Auvergne, INRAE, VetAgroSup, UMRF 0545, Aurillac, France; 10 VIB-Center of Microbiology, KU Leuven, Leuven, Belgium; 11 Nutrition and Dietetics Department, Health Sciences Faculty, Hitit University, Çorum, Türkiye; 12 Department of Food Engineering, Faculty of Engineering, Ege University, Izmir, Türkiye; 13 MED – Mediterranean Institute for Agriculture, Environment and Development & CHANGE – Global Change and Sustainability Institute, Departamento de Medicina Veterinária, Escola de Ciências e Tecnologia, Universidade de Évora, Pólo da Mitra, Évora, Portugal; 14 Institute for Biological Research ‘Siniša Stanković', National Institute of the Republic of Serbia, University of Belgrade, Belgrade, Serbia; 15 TFTAK Center of Food and Fermentation Technologies, Tallinn, Estonia; 16 INRAE, AgroParisTech, UMR1319 MICALIS Institute, Université Paris-Saclay, Jouy-en-Josas, France; 17 Biotechnical Faculty, University of Ljubljana, Ljubljana, Slovenia; 18 Research Division Microbial Food Systems, Agroscope, Bern, Switzerland; 19 Department of Epidemiology and Health Systems, Center for Primary Care and Public Health (Unisanté), University of Lausanne, Lausanne, Switzerland; 20 Université Clermont Auvergne, INRAE, UMR1019 Nutrition Humaine, Saint Genès Champanelle, France

**Keywords:** fermented food, coffee, metabolic syndrome, gut microbiota, population variability, personalized nutrition, yogurt

## Abstract

**Background:**

Diets and specific foods have a significant impact on health, and individual responses to nutritional factors vary. This variability among humans can be considered a basis for developing personalized or precision nutrition. Fermented foods (FF) contain a wide range of macro- and micronutrients, bioactive compounds, and live or dead microorganisms. FF represent a diverse range of products and have garnered significant interest due to their potential health benefits. However, consistent evidence remains limited, possibly due to heterogeneity in individual responses.

**Objectives:**

The objective of this review is to assess and compile existing evidence on the variable responses of populations to FF and to determine whether FF could be integrated into a precision nutrition strategy.

**Design:**

Interventional and observational human studies were systematically collected. The publication identified the main factors likely to contribute to variable responses to FF across all health outcomes. The question was systematically addressed to assess the available evidence and identify knowledge gaps, guiding future research. A pragmatic approach was employed, following EFSA health claim guidelines, which require an assessment of food characteristics and mechanisms of action, as well as conducting a systematic search of human interventional studies. A similar approach was used to analyze data extracted from observational studies. The population included all humans (healthy and non-healthy, of all ages), encompassing both observational and interventional studies. The intervention consisted of the ingestion of any FF, while the control was defined as the absence or lower consumption of FF or consumption of a corresponding non-FF. Outcomes included all markers of the population's health status.

**Results:**

The main factors contributing to variable responses to FF across all health outcomes were related to initial phenotypic characteristics (biological sex, geographical origin, hormonal status, and age), baseline health status [metabolic syndrome [MetS], chronic metabolic pathologies, cancer, and psychological disorders], and genetic background. Additionally, since the gut microbiota is person-specific and influences metabolic responses, particular attention was paid to its functions and role in the variability of population responses to FF.

**Conclusion:**

Collectively, this review represents a first step toward evaluating the feasibility of using FF in tailored nutritional strategies.

**Systematic review registration:**

https://osf.io/69d3f/overview.

## 1 Introduction

Fermented foods (FF) encompass a wide variety of products that differ in their raw substrates, microbial ecosystems, and bioactive compounds. They are deeply rooted in the cultural traditions of diverse populations, with variable consumption patterns (diversity, dose, and frequency). The role of FF, whether as a group of foods or individually, is actively studied concerning their effects on health (1, 2), and both the beneficial and detrimental impacts of a wide variety of FF on health are discussed in a series of reviews from the COST Action CA20128 “Promoting Innovation of Fermented Foods” (PIMENTO) (3). Beyond these global effects of diets or specific foods on population health, inter-individual differential responses to nutritional factors are now well-established (4–6). This is explained by the fact that each individual has specific and unique characteristics that lead to variable requirements or responses to diets (or more precisely, to food groups, foods, nutrients, or bioactive compounds present in the diet). This variability of response is frequently attributed to multiple factors: genetic and epigenetic background, gut microbiota composition and activity (7, 8), biological sex, race, physiological state (e.g., age, pregnancy, lactation, or growth), pathologies and history of pathologies, and numerous interconnected environmental and lifestyle parameters (e.g., physical activity, smoking, alcohol consumption, sleep, or living conditions).

Identifying the sources and causes of variability will facilitate the development of tailored nutritional strategies based on individual responses. This approach forms the foundation of precision or personalized nutrition. Within this framework, when consumed regularly, some FF can provide a wide range of macro- and micronutrients, as well as some bioactive compounds, which may help prevent the onset of metabolic dysfunctions and chronic pathologies (9). Additionally, FF, by itself or as a source of living microorganisms, could alter the host microbiota composition and activity, a potential factor contributing to inter-subject variation in response to diet (10).

However, before FF can be integrated into precision nutrition, a new field of research, it is important to assess and synthesize existing evidence on the variability of responses to FF in different populations. The field of precision nutrition has been the subject of several existing reviews, with particular emphasis on defining concepts and responding to specific nutrients in the diet or environmental factors (11–14). However, to date, no review has addressed the role of FF in this context. Our objective was to compile how populations respond to various FF, from both interventional and observational studies, to identify populations that are most responsive to specific FF or FF in general. We aimed to evaluate whether the impact of FF on various health outcomes depends on the population studied or if FF can be considered as factors capable of explaining the variability in health status within a specific population. Finally, as the gut microbiota composition is also known to be one of the major determinants of variability in response to environmental factors (including nutritional factors and, therefore, FF), the role of microbiota as a determinant of variability in response in the population is discussed.

## 2 Materials and methods

A systematic review of human studies was conducted, following the guidance of Muka et al. (15) and adhering to the requirements and checklists for conducting a scoping review (16, 17). The PROSPERO study protocol served as the basis for structuring the review process. Details of the study protocol are available under the registration “PIMENTO-SP-S6” in the Open Science Framework Registries (OSF) (https://osf.io/registries), using the Open-Ended Registration mode (18). The question raised in this study is: can the impact of FF on different health outcomes depend on specific characteristics of population groups, or, in other words, is FF a factor capable of explaining the observed variability in health status within a specific population? Consequently, can FF be recommended for specific populations (i.e., in the field of personalized-precision nutrition)?

### 2.1 Inclusion/exclusion criteria: PICO elements

The population studied in the study encompasses all humans, regardless of their health status or age. The intervention criterion corresponded to the ingestion of any of the FF contained in the PIMENTO search string (3) for FF across the following food groups: dairy, meat and fish, fruits and vegetables, beverages, legumes, cereals, and grains. Alcoholic beverages with an alcohol content of more than 1.25% were excluded. Unless specified otherwise, no limits were set for the duration or dosage of the ingested FF. Unless specified otherwise, studies investigating the application of FF other than for nutritional purposes (e.g., nasal or topical) were excluded. In addition, studies investigating probiotics were excluded unless the probiotics were added at the beginning of the fermentation process, and there were indications from the literature that the probiotic strain(s) contributed to the fermentation of the food matrix. Interventions could be designed as stand-alone interventions or as combined interventions, provided the comparator conditions are adequately controlled for in non-fermented interventions. Regarding the comparison criterion, the control group consisted of the absence of consumption, consumption of a lower amount or lower frequency of an FF, or consumption of a corresponding non-FF. Any adequate non-fermented placebo or control was also accepted as a valid comparator. Finally, the outcomes studied were the differential effects of FF on population health status. The works that have highlighted a differential health response to FF consumption (in interventional studies) or a different health status among individuals following the same diet (in observational studies) were evaluated. Of note, as there is already sufficient evidence on coffee and tea, a decision has been made to include only reviews, systematic literature reviews, meta-analyses, and studies combining large cohorts for these beverages, using the same study selection criteria as the ones described above for other FF.

### 2.2 Search strategy

The following databases were searched: Medline (from January 1970 to August 2023), Scopus (from January 1970 to August 2023), and The Cochrane Central Register of Controlled Trials (all years; The Cochrane Library). A final additional search was conducted until March 31, 2025. The generic search strings developed by the Library of the University of Zurich (Alisa Berger) for PIMENTO have been used in the literature review (PIMENTO search strings). These strings encompass terms for searching a broad scope of FF across all food groups, including all types of human studies, as well as dietary intake. The generic search string is published in the position study by Todorovic et al., to which this study protocol is linked, and is presented in supplementary data in the present study (Supplementary Table S1) (3).

### 2.3 Selection process and data extraction

The study selection has been conducted based on the following steps outlined by Muka et al. (15): step 4 (define selection criteria, following the PICO elements), step 5 (Design data collection form), step 8 (collection of references and abstracts in a single file), step 9 (elimination of duplicates), step 10 (screening of the titles and abstracts by at least two reviewers), step 11 (collection, comparison, and selection of references for retrieval), step 12 (retrieval of full text and application of selection criteria following the PICO elements), step 13, if needed (contact experts), step 14 (search for additional references), step 16 (application of the data collection form), and step 18 (preparation of the database for analysis). Of note, given the significant number of publications and the sufficient evidence on coffee and tea, it was decided to limit the inclusion of studies on these beverages to reviews, systematic literature reviews, meta-analyses, and studies combining large cohorts, using the same study selection criteria as the ones described above for other FF.

**Figure 1:**
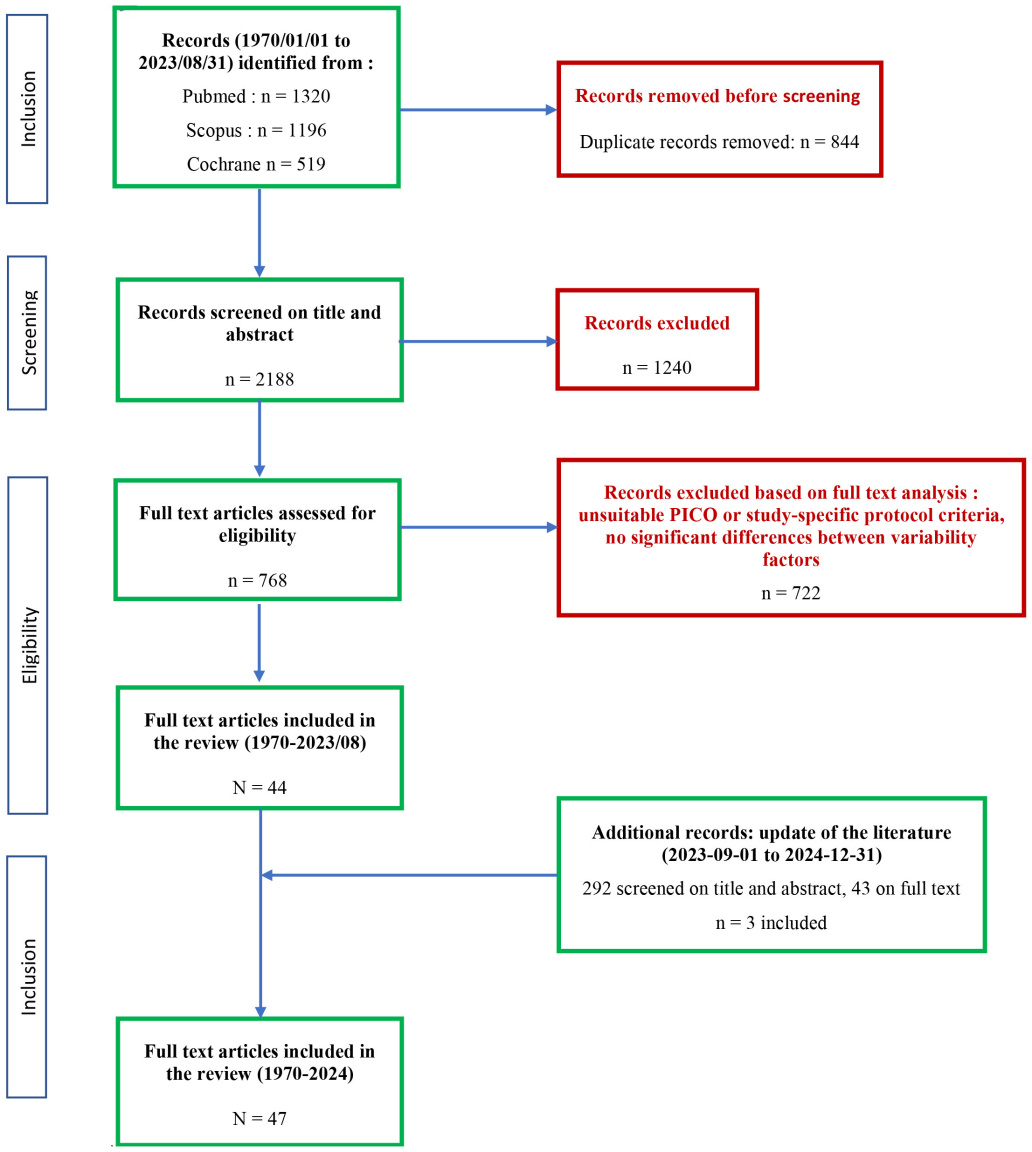
Flowchart describing the process of study selection for the present scoping review on the impact of fermented foods on health in specific populations. For details of the selection strategy and data extraction/synthesis, refer to OSF PIMENTO-SP-S6 and Todorovic et al. (3). *Of note, the articles discussed in the present study on the impact of coffee and tea on health are not included in the present selection process, as they only concern reviews (meta-analyses, systematic, and narrative reviews) on the subject (selected among the 768 full-text articles assessed for eligibility)*.

CADIMA software (19) has been used to select the studies. The selected list of studies from the three databases was uploaded to the CADIMA software, and duplicates were eliminated. A consistency check has been performed in CADIMA using titles and abstracts on a subset of the literature dataset. If necessary, the study selection process included three reviewers, rather than the two suggested by CADIMA software, to enhance the efficacy and systematicity of the reviewing process. Full texts of selected studies were uploaded automatically by the software or manually if necessary, and a second review of the studies was carried out on the full texts by at least two reviewers (as previously). An overview of the selection process for identifying relevant studies is documented in a flow diagram (Figure 1). The number of studies selected at each step of the review process is presented in Figure 1 and reflects all FF except coffee and tea, which were analyzed separately. For tea and coffee, the final number of studies selected is 22 (19 meta-analyses, two systematic literature reviews, and one analysis combining three large cohorts). In accordance with the study's inclusion and exclusion criteria, studies in which variability or sources of variability were investigated but no significant difference between the population groups was observed were also excluded.

The data extraction forms of the studies - interventional, observational studies, and review articles - were developed based on the protocol detailed in OSF PIMENTO-SP-S6. The extracted data included DOI, authors (first author and year), type of pathologies studied, specific outcomes measured, study design, country of study, sample size, and health status at baseline, FF studied, ingested dose, type of comparison made, and the effect observed (when a variability of the outcome response was significantly observed). A description of foods/constituents, health effects (with specific and well-described markers or health outcomes), and quantity of food constituent/pattern of consumption required to obtain the health effect (mentioned in EFSA guidance for presentation of health claim applications) was used as a guideline for analysis of interventional studies and mechanisms of action (20). A similar data extraction form and practical approach were used for observational studies, recognizing that the details in food characterization and composition are more limited in such studies. The two reviewers for each selected study, who had access to the full text, filled out an extraction form. The recorded data from the two reviewers for each study were compared, and discrepancies were resolved by a third reviewer as needed. No additional references were found and uploaded to the CADIMA database during the selection and data extraction processes on the full text. The data obtained were then combined and compiled in tables according to the most studied factors of variability observed in the extracted study between populations: biological sex, ethnic groups/location, age, or health status at baseline. When applicable, and within each table, stratification was made depending on the outcome studied (MetS, psychological disorders, and cancers). This allowed for a comparative and critical analysis of the data analyzed for various types of populations (and sources of variability) and the measured outcomes.

## 3 Results and discussion

We compiled interventional and observational studies. This section was structured by examining independently all major human factors likely to contribute to a significant variable response to FF across all health outcomes identified in the selected publications. These factors were divided as follows: initial global phenotypic characteristics (e.g., biological sex, geographical origin, hormonal status, age), health status at baseline [e.g., metabolic syndrome (MetS), insulin, cancer, psychological disorders], genetic background, and other sources of variability (gut microbiota, the “responders” concept). As the composition and characteristics of FF are essential for evaluating the health effects of FF consumption, this topic was also addressed in a dedicated section below.

### 3.1 Some global phenotypic characteristics as a source of variability

#### 3.1.1 Biological sex is rarely studied by itself

Among the biological sources of variability in responses to FF consumption, biological sex is one of the most studied, with 23 publications identified from the literature search (Table 1). The majority of the articles were from observational studies, with only two articles focusing on interventions for MetS. Diverse outcomes have been studied, ranging from MetS, associated parameters and metabolic pathologies (*n* = 9, BMI, type 2 diabetes (T2D) blood lipids, blood pressure) (21–29), to psychological disorders (*n* = 6) (30–35), cancer (*n* = 4, colorectal, gastric, bladder) (36–39), gut microbiota (*n* = 1) (40), or aging (*n* = 2) (41, 42). These studies were conducted in populations from different origins, with mostly one article per country (North of Europe: Norway, UK, Netherlands, Denmark, Poland; South of Europe: Spain; Asia: Japan, Korea; South America: Uruguay; Oceania: Australia). There were two articles implemented in Finland, Sweden, and France, and four in the USA. The sample sizes of the populations studied in these publications also varied, ranging from tens of volunteers in interventional studies to cohorts of nearly 100,000 participants. In terms of FF studied, the majority of them were dairy products, with yogurt being the most studied FF.

**Table 1:** FF intake (except for tea and coffee) and analysis of the variability of responses between females and males in various physio-/pathological situations: MetS, psychological disorders, cancers, gut barrier markers, and muscle status.

**Reference**	**Specific outcome measured**	**Study design**	**Country studies**	**Sample size**	**FF studied**	**Comparison**	**Main effect/Variability observed**
**Metabolic syndrome**							
Chen et al. (2014) (21) doi: 10.1186/s12916-014-0215–1	T2D risk	Observational—longitudinal [3 cohorts (follow-up studies): HPFS (males), NHS (females) and NHSII (females)] (20–30 years of follow-up)	USA	subjects (67,138 females in NHS, 85,884 females in NHS II, 41,436 males in HPFS) globally healthy	Dairy (yogurt and cheese)	Lowest quintile (lowest serving) to highest quintile, occurrence of T2D over time	Yogurt: Beneficial in reducing the risk of developing type 2 diabetes in females (NHS cohorts). Neutral for males (HPFS cohort)
Johansson et al. (2018) (22) doi: 10.1186/s12937-018-0418-y	Anthropometry (BMI), blood pressure, serum lipids (total cholesterol, total triglycerides, HDL, and LDL cholesterol), and blood glucose evolution	Observational—longitudinal cohort (25 years of follow-up)	Sweden	90,512 volunteers (46,462 females, 44,050 males)	Dairy (fermented milk, cheese, and butter)	Lowest quintile	Cheese: Inversely associated with undesirable S-triglyceride levels and undesirable fasting B-glucose levels in females Butter: Inversely associated with undesirable fasting B-glucose levels in males
Mennen et al. (1999) (23) doi: 10.1016/s0049-3848(99)00027-4	Tissue-type plasminogen activator antigen (t-PA-Ag) level	Observational—cross-sectional	France	594 volunteers (295 females, 299 males)	Dairy (cheese)	Lowest quartile (0 portion a day)	Cheese: decreased tPA-Ag levels were observed with increased cheese consumption in females, with a similar trend noted in males
Vergnaud et al. (2008) (24) doi: 10.3945/ajcn.2007.25151	Body weight and waist circumference evolution	Observational—longitudinal (6 y follow-up)	France	2,267 volunteers (1,022 females, 1,245 males)	Dairy (cheese and yogurt)	Lowest quartile	Yogurt: overweight males: decrease in weight change and waist circumference (also observed for milk) Normal-weight females: tendency toward a positive association with weight change Cheese: no association observed, regardless of sex or weight status
Zykova et al. (2015) (25) doi: 10.1186/s12937-015-0032-1	Serum uric acid (SUA) level	Observational—cross-sectional	Australia	Caucasian adults (9,734, 5,439 females, 4,295 males)	Dairy (Yogurt and cheese) Cereals (bread)	Lowest to highest consumption	High yogurt consumption: Strong inverse relationship with SUA in obese and non-obese females and in obese males, but no relationship in obese males High-fiber bread: Associated with lower SUA in non-obese males and obese females (trends in other groups)
Zykova et al. (2015) (25) doi: 10.1186/s12937-015-0032-1	SUA level	Observational—cross-sectional	Norway	Caucasian adults (3,034, 1,471 females, 1,560 males)	Dairy (Yogurt and cheese) Cereals (bread)	Lowest to highest consumption	Bread and cheese: Strong inverse relationship with SUA in all groups except non-obese females High-fiber bread: Associated with lower SUA in non-obese males
Ericson et al. (2015) (26) doi: 10.3945/ajcn.114.103010	T2D risk	Observational—longitudinal (14 y follow-up)	Sweden	26,930 (approximately 60% females)	Dairy (fermented milk, cheese, butter) beverage (chocolate)	Lowest quintile (lowest intake)	Cheese: inverse association with T2D risk in females Except for the interaction between cheese intake and sex [also reflected in the interaction between high-fat fermented dairy product intake and sex (*P* = 0.01)], no other significant interactions were found between any other examined food intake and sex
Lacoppidan et al. (2015) (27) doi: 10.3390/nu7105418	T2D risk	Observational—longitudinal (15.3 y follow-up)	Denmark	55,060 volunteers (28,953 females, 26,107 males)	Cereals (rye bread)	Below or above a median value of intake for rye bread	Decreased risk of T2D in males when consuming rye bread >the median, regardless of the model of adjustment used Less the case in females (only true for model 2)
Leinonen et al. (2000) (28) doi: 10.1093/jn/130.2.164	Serum total, HDL, and LDL cholesterol, Triacylglycerol	Interventional: 4 weeks treatment/crossover/4 weeks washout	Finland	40 volunteers (18 males, 22 females)	Cereals (rye bread)	Replacement of customarily used breads by rye bread or wheat bread (20% daily energy intake)	Females: no effect on all parameters tested males: decreased serum total cholesterol (dose-dependent effect)
Dugan et al. (2016) (29) doi: 10.1080/07315724.2015.1022637	MetS parameters: inflammatory markers (adipokines, chemokines, glycoproteins, C-reactive protein—CRP, gene expression in peripheral blood mononuclear cells, hepatic enzymes, intracellular and vascular adhesion molecules (ICAMs and VCAMs)	Interventional/6 weeks/crossover	USA	37 volunteers (24 females, 13 males)/Met S at baseline	Dairy [mixture of milk, low-fat yogurt, and cheese (three dairy servings/day)]	Energy-matched control (granola, juice)	Dairy mixture (mainly fermented): Decreased BMI, body weight, and waist circumference in females but not males In females, decreases in CRP, MCP-1, and TNF-α were observed, with a trend toward reduced leptin levels. Many of these parameters (CRP, leptin, and MCP-1) were higher at baseline in females compared with males No impact of treatment on plasma inflammatory marker levels in males
**Psychological disorders**							
Sun et al. (2020) (30) doi: 10.1016/j.jad.2020.05.095	Depressive symptoms	Observational—cross-sectional	USA	21,924 males and females, 11,140 males, 10,784 females	Dairy: yogurt and cheese	From the lowest quartile (0 intake)	Yogurt: decreased depressive symptoms in females Notably, low-fat milk and moderate cream intake were associated with decreased depressive symptoms
Rintamäki et al. (2014) (31) doi: 10.3109/08039488.2013.851736	Depression, anxiety, and alcohol use disorders	Observational—cross-sectional	Finland	5,504 subjects (3,009 females and 2,495 males)	Dairy (cheese, fermented milk products) cereals (rye and wheat), beverages (coffee and tea)	From a healthy population	Fermented milk product: decreased fermented milk product intake in females with alcohol use disorders (NS in males) Cheese: decreased cheese intake in females with anxiety disorders (NS in males) Tea: increased tea intake in males with anxiety disorders No conclusions on chocolate (particularly in males) when combined with sweets
Perez-Cornago et al. (2016) (32) doi: 10.3945/jn.116.233858	Depression risk	Observational—longitudinal (9.3 y follow-up)	Spain	14,539 volunteers (55 to 60 % females)	Dairy (Yogurt)	Lowest quartile (< 0.5 serving/week)	Yogurt: positive association between increased low-fat yogurt intake and depression risk, significant only in females Whole-fat yogurt is associated with a lower incidence of depression in females Nothing significant in males
Mahdavifar et al. (2022) (33) doi: 10.1080/1028415X.2021.1969065	Psychological disorders: depression, anxiety, stress	Observational—cross-sectional	Iran	7,387 (49.7% males, 51.3% females)	Dairy [Yogurt, high-fat yogurt, cheese, yogurt drink, kashk (cheese)]	Lowest tertile	Yogurt drink: negative association with depression in males, not females, particularly at high intakes The same for anxiety in males, with a mild beneficial effect of total yogurt in females No impact of fermented dairy on stress in both males and females
Murai et al. (2022) (34) doi: 10.1007/s00394-022-02937-5	Disabling Dementia	Observational—Longitudinal (10-year follow-up)	Japan	41,447 (22,456 females, 18,991 males)	Legumes (Natto, Miso)	Lowest quintile	Natto: inverse relationship with dementia in females under 60 years of age. Not in males Miso: tendency for inverse relationship with dementia in females under 60 years old. Not in males
Jamka et al. (2024) (35) doi: 10.3390/nu16050644	Mild cognitive impairment in 50–70 y.o. population	Observational—cross-sectional	Poland	190 volunteers (143 females and 47 males)	Butter, fermented milk beverages, white bread	No consumption of the product	Significant differences in the frequency of butter intake (*P* = 0.0173) and fermented milk beverages (*P* = 0.0235) between “mild cognitive impairment” and “normal cognitive function” subjects were observed only in females. Different patterns of intake between the two groups. Not observed in males. No differences for breads
**Cancers**							
Pala et al. (2011) (36) doi: 10.1002/ijc.26193	Colorectal cancer risk	Observational—longitudinal (12-year follow-up)	Italy	45,241 (31,063 females, 14,178 males)	Dairy (Yogurt)	Lowest tertile	The protective effect of yogurt in males (regardless of the model) is more pronounced in females However, there was a significant interaction of sex on the association between yogurt consumption and CRC risk (whatever the model used)
Keszei et al. (2010) (37) doi: 10.1093/aje/kwp399	Bladder cancer risk	Observational—longitudinal (control + cases) (16.3 y follow-up)	The Netherlands	5,781 (3,357 males and 2,424 females)	Dairy (fermented milk, cheese, and butter)	Lowest quintile	Butter: positive association (increased risk) in females No association observed in males
Deneo-Pelegrini et al. (2002) (38) doi: 10.1097/00008469-200208000-00009	Colon and rectal cancer risk	Observational—cross-sectional (control + cases)	Uruguay	1,936 volunteers (1,176 males, 760 females)	Cereals (white bread)	From the lowest quartile	Bread: positive association (increased risk) with colorectal cancer in females, NS in males Of note: plant foods and all vegetables and fruits are protective for males only
Hara et al. (2012) (39) doi: 10.3945/ajcn.111.020479	Gastric cancer	Observational—longitudinal (5-year follow-up)	Japan	84,781 volunteers (39,569 males and 45,312 females)	Legumes (Miso soup)	From the lowest quartile	In males: no impact of miso soup consumption on gastric cancer risk (whatever the location), after adjustment for BMI, smoking status, ethanol intake, family history of gastric cancer, vegetable intake, fruit intake, fish intake, salt intake, and total energy intake In females: tendency for an inverse association with the risk of gastric cancer (but not upper third and distal cancers when taken separately), when adjusted for the same parameters as males Not true for other soy products
**Gut barrier markers**							
Luo et al. (2021) (40) doi: 10.1007/s00394-020-02303-3	Soluble CD14 (marker of gut barrier dysfunction)	Observational—cross-sectional	USA (two cohorts: one woman and one man)	1,076 (632 females, 444 males)	Dairy (Yogurt)	Non-consumers (+3 additional levels of consumption)	In the males's cohort: inverse association with sCD14 (attenuated when adjustments are made for confounders in models) In the female's cohort, no significant effect was observed, despite a similar inverse association being noted
**Muscle status**							
So and Joung (2020) (41) doi: 10.3390/nu12092537	Risk of developing a low skeletal muscle mass index	Observational—longitudinal (12 y follow-up)	Korea	4,412 volunteers (2,096 males, 2,316 females)	Dairy (Yogurt)	Lowest tertile	Higher dairy protein consumption globally is associated with a lower risk of having a low muscle index in males, not females (this is also true for yogurt specifically) Higher milk intake in global dairy regions is associated with a lower risk of having a low muscle index in males, not females. Not true for yogurt intake (NS for both sexes)
Gedmantaite et al. (2020) (42) doi: 10.1016/j.mad.2020.111269	Handgrip strength	Observational—cross-sectional	UK	68,002 subjects (34,187 females, 33,815 males)	Dairy (cheese) Cereals (bread)	Linear regression associations	Cheese: negative association with grip strength in females, not males Bread: negative association with grip strength in males, not females

Focus on observational and interventional studies.

We noted that biological sex is rarely, if ever, studied in isolation, and variability of response attributed to biological sex is generally not considered a primary outcome. It's generally used as an adjustment parameter, together with BMI, for instance (29). It serves as a means to highlight the significant effects of FF on targeted outcomes when variability within cohorts is too great to allow for the measurement of a significant effect of FF at the overall population level. The consequence of this is that several biases can occur: comparison between males and females is conducted between cohorts that can be different also based on other parameters [e.g., the Health Professionals Follow-up Study (HPFS) cohort includes males, while the Nurses' Health Study (NHS) cohorts include females (21)]; inclusion of populations from distant places with varied nutritional habits and lifestyles, for example, Norway and Australia (25). Additionally, baseline health or physiological characteristics (e.g., BMI) and lifestyle habits (e.g., alcohol consumption and smoking) often differ between males and females, with females generally exhibiting higher baseline inflammation levels. This may explain some differences that are not strictly sex-related *sensu stricto* [e.g., see (24, 41)]. Therefore, it remains challenging to establish a general pattern for the role of sex in the differential responses observed.

However, a few sex-related patterns did emerge from our literature review results (Table 1). For metabolic outcomes, in females, yogurt consumption was positively associated with a reduced risk of MetS, a decreased BMI (21), lower inflammation (29), and weight loss (24) in normal-weight individuals. In males, it was associated with decreased weight in overweight people (24). Cheese consumption was associated with improvements in the lipid and glucose profiles, as well as a decrease in cardiovascular risk, based on various biomarkers across different studies (22, 23), although this association was not consistently observed [e.g., see (24)]. However, butter consumption was associated with lower blood glucose levels only in males (22). Bread consumption had a small effect on the serum uric acid (SUA) levels in females, while it decreased lipids and SUA in males in Norway (no effect on SUA in Australia) and had a general positive effect on type 2 diabetes (T2D) in males (25).

For psychological disorders, in females, a contrasting effect of yogurt according to fat content was observed, with a negative effect of low-fat yogurt consumption on depressive symptoms (32), whereas whole-fat yogurt was beneficial. In the same study, no effect of yogurt, regardless of fat content, was observed in males. However, in another study, yogurt was demonstrated to be associated with decreased depression and anxiety in males (with a mild effect on anxiety in females) (33). Butter and fermented milk beverage consumption patterns were also modified in females with mild cognitive impairment compared to females with normal cognitive functions (35). This was not the case for males. Finally, natto consumption was associated with decreased dementia in females only (34).

In the field of cancer research, yogurt was associated with a decreased colorectal cancer development in males only (36), whereas butter was linked to an increased risk of bladder cancer in females only (37). In females, bread consumption was associated with an increased risk of colorectal cancer (38), and miso soup with a decreased gastric cancer risk (39).

Finally, in musculoskeletal health studies, yogurt intake was associated with an increase in muscle mass in males (41). Conversely, cheese consumption was associated with a decrease in handgrip strength in females, and bread consumption was associated with a decrease in handgrip strength in males (42).

Based on these data, variability in responses between males and females has been demonstrated across various studies and for various outcomes. However, it is also clear that the variability is rarely consistently confirmed. One suggestion, considering the studies highlighting a sex differential response to FF (observed frequently in dairy foods in observational studies), is that additional studies with a higher number of participants and increased precision in FF intake assessment, as well as identification and adjustment for potential sex-specific confounders, are needed. Indeed, even in studies examining MetS parameters (the most extensively studied outcome), the number of publications is too low to draw a definitive conclusion. This is further explained in the corresponding articles: when studied, a significant sex-specific response is not always consistently observed; when such a response exists, it may fail to reach significance due to an inadequate control for potential confounding factors. This was also observed in the present work, even if the studies were discarded from the search because they were non-significant, for example, see (43) on MetS or (44) on all causes of mortality.

Although a few underlying mechanisms of action may contribute to the observed sex differences, the majority of explanations found in the literature relate to potential confounding factors. These include incomplete evaluation of alcohol consumption [that can be higher in males but not necessarily estimated (40, 41)], BMI at baseline (27), differences of intakes (in nature and quantity) between sexes, general inadequate evaluation of the FF intake (and impossibility to evaluate differences between males and females on this parameter), and undiagnosed pathologies or metabolic shifts at baseline, including microbiota dysbiosis (32). Only a few studies have mentioned that some sex differences in metabolism could explain the differential response to FF on various health outcomes, including higher susceptibility of females to inflammation-related tumors (36); differential calcium homeostasis in females due to sex hormones (37); and differences in rectal mucosa between males and females (38). However, even in these latter studies, the potential mechanisms of action remain hypothetical and have not been tested.

#### 3.1.2 Geographical origins resulted in different responses to dairy and bread

Three studies (two observational and one interventional) demonstrated a differential response to FF in populations from various geographical origins, as well as between black and white populations, or Caucasian/non-Caucasian populations (Table 2). The observational studies included a cross-sectional study by Zykova et al. (25), conducted in Australia and Norway, and a case-cohort study by Rosen et al. (47) in the USA, both of which focused on pregnant females with singleton pregnancies. The interventional study by Wolever et al. (45) conducted in Canada compared Caucasian/non-Caucasian populations. Finally, a meta-analysis compared the impact of yogurt consumption on colorectal cancer incidence, using data from studies conducted in various countries worldwide (including America, Asia, Africa, and Europe) (46). Here, in addition to ethnicity, variability between obese and non-obese subjects, as well as between males and females subjects, was also examined simultaneously. The studies investigated yogurt, cheese, and/or bread, assessed either through dietary assessments or direct interventions. Key outcomes varied widely between these studies, ranging from SUA levels, glycemic response, and vaginal microbiota composition (and associated risk of pregnancy outcome) to colorectal cancer.

**Table 2:** FF intake (except for tea and coffee) and analysis of the variability of response between ethnic group/location of populations in various physio/pathological situations: MetS, cancers, and pregnancy-associated pathologies.

**Reference**	**Specific outcome measured**	**Study design**	**Country studies**	**Sample size**	**FF studied**	**Comparison**	**Main effect/Variability observed**
**Metabolic syndrome**							
Zykova et al. (2015) (25) doi: 10.1186/s12937-015-0032-1	Serum uric acid (SUA) level	Observational—cross-sectional	Australia/Norway	Australia: Caucasian adults (9,734, 5,439 females, 4,295 males) Norway: Caucasian adults (3,034, 1,471 females, 1,560 males)	Dairy (Yogurt and cheese) cereals (bread)	Lowest to highest consumption	Yogurt: Inverse relationship with SUA in the Australian cohort (except obese males). No effect in the Norwegian cohort Cheese: Inverse relationship in both cohorts (except non-obese Norwegians) High-fiber bread: Associated with lower levels of SUA in non-obese Norwegian and Australian males, and obese Australian females. Similar trends in other groups
Wolever et al. (2009) (45) doi: 10.1038/ejcn.2009.30	Incremental area under the curve (AUC) of glycemic response	Interventional—randomized three blocks (1 test food and glucose) (randomized within and between blocks)	Canada	77 healthy participants (40 Caucasian/37 non-Caucasian)	White bread (50 g available carbohydrate)	Control: glucose (50 g)	White bread: AUC for glucose was greater in non-Caucasians than in Caucasians after a white bread meal. No differential response with the other foods tested (chocolate-chip cookies, fruit leather, or glucose)
**Cancer**							
Sun et al. (2021) (46) doi: 10.3389/fnut.2021.789006	Colorectal cancer incidence	Meta-analysis/16 observational studies (cross-sectional, cohort, and case-control)	NA	1.129.035 participants, eight studies in Europe, three in North America, two in Asia, two in Africa, one in various European countries	Yogurt	Lower to higher consumption	Yogurt consumption is associated with a lower risk of cancer in Europe and Africa. Not the case for Asia and North America
**Pregnancy pathologies**							
Rosen et al. (2022) (47) doi: 10.1111/ppe.12830	Vaginal microbiome associated with pregnancy outcome	Observational case-cohort study	USA	Healthy pregnant females with singleton pregnancies, Black and white (634 participants, 264 Black, 370 white)	Yogurt	Lowest quartile consumption	Yogurt: yogurt intake is associated with increased likelihood of the predominant bacteria species in the vagina being *Lactobacillus crispatus* vs. *Lactobacillus iners* (lower risk of preterm birth) in black females only. However, no statistical differences were observed between groups

Focus on observational, interventional studies, and meta-analysis.

Zykova et al. (25) highlighted distinct responses between Norwegian and Australian populations to yogurt. A more favorable effect observed in the Australian cohort was most likely attributed to a more extensive consumption of live probiotic bacteria in yogurt preparations compared to Norway at baseline, suggesting that the difference in response between the two cohorts was more linked to variation in nutritional habits than to an impact of ethnicity or location of the study *per se*. Differential responses were also observed following the consumption of high-fiber bread (Table 2). Similar to other studies, dietary habits were suggested as a possible factor in differential responses, with lower fat intake reported in the Norwegian cohort, which may influence intestinal lipid absorption mediated by dietary fiber. In the same line, the fact that yogurt consumption was associated with a lower risk of cancer in Europe and Africa but not in Asia and North America was not due to ethnicity or location *per se* but to the imbalance in the number of studies carried out in the various continents (more studies in Europe) and variable nutritional habits (and the quantity of yogurt ingested) between countries (46).

The Rosen et al. (47) study showed that yogurt consumption was linked to a higher likelihood of harboring a beneficial *Lactobacillus crispatus* vagitype rather than *Lactobacillus iners*, resulting in a lower risk of preterm birth. While slightly stronger associations were found among Black females, the overall effects were not significantly different between white and black females. When Wolever et al. (45) examined the blood glucose area under the curve and the glycemic index in response to white bread intake, the effects were significantly different across the ethnic groups. More specifically, non-Caucasians exhibited a greater glycemic response and a higher glycemic index compared to Caucasians, suggesting possible genetic differences in salivary amylase gene copy number. This gene variation, associated with increased salivary amylase activity, may impact starch digestion in the mouth and potentially gastrointestinal (GI) function.

#### 3.1.3 Hormonal state: the case of pre- and post-menopause

In our literature search, we did not find studies that showed a significant differential health status response to FF in females populations during pre- and post-menopause. The outcomes generally studied in this field are hormone-related cancers or osteoporosis. For these outcomes, some studies failed to show variable responses following higher consumption of FF [e.g., fermented soy paste associated with reduced risk of breast cancer in both pre- and post-menopausal females (48)]. In other cases, data were too scarce (publications available only as an abstract) to evaluate whether risk factors for developing osteoporosis varied between pre- and post-menopausal females following the intake of FF (namely, hard cheese and yogurt) (49). However, some studies have shown a differential response depending on menopausal status following coffee intake (50) (described in part 3.3.1).

#### 3.1.4 Age, only a reflection of health status?

Similar to biological sex, age is commonly examined as a clustering variable, often in conjunction with BMI, dietary patterns, and health conditions. Among the studies identified in our search and evaluating age as a potential source of variable response to FF, only three reported a significant differential health response to FF depending on age (Table 3). Two observational studies investigated the impact of fermented dairy products (yogurt and cheese) and legumes (natto and miso) on psychological disorders, respectively (30, 34). Sun et al. (30) have shown a negative association between yogurt intake (< 131 g/day) and depressive symptoms in populations 60 years old and above, which was not the case for younger populations. Murai et al. (34) showed a sex/age combined effect, reporting an inverse relationship (significant or trending) between the risk of developing dementia and the intake of natto and miso in females under 60 years of age. This was not the case in males of all ages and females above 60 years old.

**Table 3:** FF intake (except for tea and coffee) and analysis of the variability of response according to the age of the individuals in various physio/pathological situations: MetS and psychological disorders.

**Reference**	**Specific outcome measured**	**Study design**	**Country studies**	**Sample size**	**FF studied**	**Comparison**	**Main effect/Variability observed**
**Metabolic syndrome**							
Ried et al. (2017) (51) doi: 10.1002/14651858.CD008893.pub3	Blood pressure	Meta-analysis of RCTs (35 trials): effect of chocolate or chocolate products on systolic and diastolic blood pressure in adults	NA	1,804 participants	Chocolate or chocolate products	No cocoa or cocoa product (placebo) or lower levels	Cocoa: the effect could be slightly attenuated by age, such that blood pressure reduction tended to be greater in younger individuals (mean age range, 18 to 49 years; 18 trials) compared with older individuals (mean age range, 50 to 73 years; 20 trials)
**Psychological disorders**							
Sun et al. (2020) (30) doi: 10.1016/j.jad.2020.05.095	Depressive symptoms	Observational—cross-sectional	USA	21,924 males and females, 11,140 males, 10,784 females	Dairy: Yogurt and cheese	From the lowest quartile (0 intake)	Yogurt: Negative association between Yogurt intake (< 131 g/day) and depressive symptoms in populations 60 years old and above Not significant for individuals aged 18–39 years old Not significant in the majority of adjusted models for 40–59-year-olds
Murai et al. (2022) (34) doi: 10.1007/s00394-022-02937-5	Disabling Dementia	Observational—Longitudinal (10-year follow-up)	Japan	41,447 (22,456 females, 18,991 males)	Legumes (Natto and Miso)	From the lowest quintile	Natto: Inverse relationship with dementia in females under 60 years old. Not in males under 60 years, nor males and females aged 60 years and older Miso: Tendency for inverse relationship with dementia in females under 60 years old. Not in males under 60, nor males and females aged 60 years and older

Focus on observational, interventional studies, and a meta-analysis.

The proposed mechanisms of action by the authors include the presence of macro- and micronutrients in fermented dairy products and nattokinase in natto [e.g., see (52)] as possible explanations for the beneficial effects of the two categories of FF on psychological disorders. However, no explanation is provided or hypothesized to explain why females may be more responsive to these foods than males. Finally, a Cochrane meta-analysis examining the impact of chocolate and chocolate products on blood pressure found a slightly lower beneficial effect of cocoa on blood pressure reduction in populations aged 50–73 years compared to those aged 18–49 years (51). These data come from short-term interventional studies only. The lower response to cocoa with age was biologically explained by an age-related increase in arterial stiffening. In the short-term studies included in the review, an age-related decrease in vascular reactivity could limit the capacity to respond to the physiological stimulus (chocolate and its associated flavanols).

We noticed that age and stage of life are among the most studied sources of variability factors and are often considered potential confounding factors (data generally available in cohorts) that can be responsible for differential responses to food intake or metabolic challenges (5). This can be explained by age-related decreases in metabolic flexibility in response to the metabolic challenge that meal intake represents (53). In females, menopause can also overlap with (and be partially the cause of) this loss of flexibility. Surprisingly, very few studies have demonstrated significant differential responses to FF depending on the age of individuals, possibly because of the narrow age range of subjects enrolled in interventional studies. This can make sense if one considers the fact that, beyond age, the health status of each individual is more responsible for the degree of response of physiology and metabolism to a nutritional challenge. The variability of response in a category of age (e.g., above 60 years old) with variable health status may obscure any potential age-related differential response to nutritional challenges, including FF. However, differential age-dependent regulation of the circulatory levels of metabolites (some of which serve as markers or sentinels of our health status) was observed following the ingestion of a challenge meal (yogurt) (54). A more detailed study would be worthwhile since age has one of the highest potentials for further development of more targeted nutrition recommendations.

### 3.2 Health status at baseline

A total of 16 publications [six observational cohorts, six interventional studies (one study split into fasted and fed states), and four systematic literature reviews and meta-analyses] have been identified using our search protocol for their differential response to FF depending on health status at baseline (Table 4). A majority of studies deal with MetS and/or metabolic disruptions or pathologies; one concerns depression/anxiety, and one concerns upper erodigestive tract cancer. Concerning MetS, the specific outcomes studied include serum lipids, biomarkers or factors associated with MetS or cardiovascular pathology risks, body weight, and inflammation/oxidative stress in relation to fermented dairy intake (24, 25, 56–58, 60). Another set of interventional studies focused on understanding the variability of glycemic responses (blood glucose and insulin responses) within a population to breads made from refined or wholemeal flours (59, 61, 62). A set of publications focused on the role of cocoa, chocolate, or chocolate products and their impact on blood pressure (66). Fermented soy products and apple cider vinegar were also studied for their impact on serum lipids and glycemic parameters (55, 65). Finally, the role of fermented dairy was evaluated for its impact on depression and anxiety (31) and upper erodigestive tract cancer risk (68).

**Table 4:** FF intake (except for tea and coffee) and analysis of the variability of response depending on health status at baseline and/or metabolic-immune profile-biomarker levels in various physio/pathological situations: MetS, psychological disorders, and cancers.

**Reference**	**Specific outcome measured**	**Study design**	**Country studies**	**Sample size**	**FF studied**	**Comparison**	**Main effect/Variability observed**
**Metabolic syndrome**							
Zykova et al. (2015) (25) doi: 10.1186/s12937-015-0032-1	Serum uric acid (SUA) level	Observational—cross-sectional	Australia/Norway	Australia: Caucasian adults (9,734, 5,439 females, 4,295 males) Norway: Caucasian adults (3,034, 1,471 females, 1,560 males)	Dairy (Yogurt and cheese) Cereals (bread)	Lowest to highest consumption	Yogurt: inverse relationship with SUA in the Australian cohort (except obese males). No effect in the Norwegian cohort Cheese: inverse relationship in both cohorts (except non-obese Norwegians) High-fiber bread: associated with lower levels of SUA in non-obese Norwegian and Australian males and obese Australian females. Similar trends in other groups.
Wilunda/2020 (55) doi: 10.1007/s00394-019-02057-7	Serum lipids and glycohemoglobin (HbA1c)	Observational—Longitudinal (5 y follow-up)	Japan	7,252 volunteers (4,860 females, 2,392 males)	Fermented soy, miso, and natto	Lowest quartile	Fermented soy: BMI ≥25: inverse relationship for total cholesterol, non-HDL cholesterol, and LDL cholesterol. NS for a BMI of < 25 Natto: BMI ≥25: inverse relationship for total cholesterol, non-HDL cholesterol, LDL cholesterol, and triglycerides. NS for a BMI of < 25 No relationship for non-fermented soy, total soy food, and miso
Vergnaud et al. (2008) (24) doi: 10.3945/ajcn.2007.25151	Body weight and waist circumference evolution	Observational—longitudinal (6 y follow-up)	France	2,267 volunteers (1,022 females, 1,245 males)	Dairy (cheese and yogurt)	Lowest quartile	Yogurt: Overweight males: decrease in weight change and waist circumference (also true for milk) Normal-weight females: tendency for positive association with weight change Cheese: No association regardless of sex and weight
Rosell et al. (2006) (56) doi: 10.1093/ajcn/84.6.1481	Body weight change over the follow-up.	Observational—longitudinal (7–10 years)	Sweden	19,325 perimenopausal females	Dairy (cheese and butter)	< 1 serving/day (vs. ≥1 serving/day)	Cheese (but also whole milk and sour milk) is inversely associated with weight gain for a consumption of ≥ one serving/day For females with a BMI of < 25, milk, sour milk, and cheese are inversely associated with weight gain for a consumption of ≥ one serving/day For females with a BMI of ≥25, only cheese is inversely associated with weight gain for a consumption of ≥ one serving/day
Pei et al. (2017) (57) doi: 10.1017/S0007114517003038 Pei et al. (2018) (58) doi: 10.1093/jn/nxy046	Biomarkers of inflammatory status and endotoxin exposure	Interventional (9-week treatment)	USA	120 premenopausal females, obese and non-obese. Randomized in four groups of 30 volunteers (yogurt non-obese—YN, Yogurt obese—YO, control non-obese—CN, control obese—CO)	Low-fat Yogurt (339 g/day) Challenge postprandial state (Pei, 2018); 226 g yogurt	Soya pudding (324 g/day) Challenge post-prandial state (Pei, 2018); 216 g soya pudding	Yogurt: Pei 2017: Fasted state Global effect: decreased biomarkers of chronic inflammation (TNF-α/sTNFR-RII) and endotoxin exposure (increased plasma IgM EndoCAb, decreased LBP/sCD14) Differential effects between obese and non-obese volunteers: YO peripheral blood mononuclear cells expression of NF-κB inhibitor α and transforming growth factor β1 increased vs. CO at 9 weeks
							Lower diastolic blood pressure in the YO group vs. other groups (treatment x obesity: *P* = 0.09) YO: Higher 2-arachidonoylglycerol plasma levels (improvement of intestinal barrier) vs. CO Pei 2018: postprandial state Global effect: YN and YO had ≥40% lower net iAUC of LBP-to-sCD14 ratio and plasma IL-6 concentration than CN and CO, respectively 1 AUC of LBP-to-sCD14 ratios of YO and YN were less than half of those of the control groups Differential effects between obese and non-obese volunteers: Premeal yogurt consumption led to 72% higher net iAUC of sCD14 in the O group than consumption of the control snack, whereas the *N* groups were not different between treatments (treatment x obesity: *P* = 0.03) Postprandial hyperglycemia was not different between CO and YO; in contrast, YN had 57% less postprandial hypoglycemia than did CN (*P*-interaction = 0.0013)
Korem et al. (2017) (59) doi: 10.1016/j.cmet.2017.05.002	Glycemic response/post-prandial glycemic response to bread	Interventional—crossover (1-week dietary intervention, 2-week washout)	Israel	20 volunteers (nine males, 11 females)	Sourdough bread and white bread	Comparison of the response to the two sources of bread, before and after each dietary intervention	Bread: No difference in terms of oral glucose tolerance test (OGTT) response and wake-up glucose levels to the two breads, before and after nutritional intervention One-week bread consumption (regardless of the bread type) alters clinical parameters (iron, magnesium, calcium, hepatic enzymes, CRP, and cholesterol) without altering gut microbiota composition However, a high interpersonal variability is observed in the postprandial glycemic response (PPGR). The PPGR to breads for each volunteer can be predicted by using microbiota features (and particularly the abundance of microbiome species, such as *Coprobacter fastidiosus* and *Lachnospiraceae bacterium 3_1_46FAA*).
Ito et al. (2017) (60) doi: 10.3920/BM2016.0102	Serum low-density lipoprotein (LDL)-cholesterol, malondialdehyde-modified low-density lipoprotein (MDA-LDL), MDA-LDL/LDL-cholesterol, blood pressure	Interventional, randomized, placebo-controlled trial (preconsumption 4 weeks, consumption 12 weeks, postconsumption 4 weeks)	Japan	59 volunteers (30 placebo group, 29 intervention group)	*S. thermophilus* YIT 2001 fermented milk (ST group)	Comparison with placebo (non-fermented milk) (C group)	Yogurt: Global effect: the ST group had significant reductions in MDA-LDL, MDA-LDL/LDL-cholesterol, systolic blood pressure (SBP), and diastolic blood pressure (DBP) vs. the C group during the consumption period Stratified analysis: Reductions in MDA-LDL, MDA-LDL/LDL-cholesterol, SBP, and DBP in the ST group compared with the PC group during the consumption period in subjects who had above median (65 U/L) levels of oxidative stress marker MDA-LDL at baseline, but not subjects with levels below the median
Moazzami et al. (2014) (61) doi: 10.3945/jn.113.188912	Post-prandial insulin response to test meals (including breads) + other serum metabolites	Interventional, randomized, crossover (test meals, no adaptation to diets)	Finland	20 volunteers (post-menopausal females)	Refined wheat bread, whole-meal rye bread, refined rye bread	Refined wheat bread is considered the control	Bread: Global effect: eight amino acids responded differently postprandially following the ingestion of different breads Stratification: association between metabolic profile at baseline and post-prandial serum insulin levels: Females with higher fasting concentrations of leucine and isoleucine and lower fasting concentrations of sphingomyelins and phosphatidylcholines had higher insulin responses despite similar glucose concentration (for all breads)
Kovatcheva-Datchary et al. (2015) (62) doi: 10.1016/j.cmet.2015.10.001	Glucose metabolism	Interventional, randomized, crossover (3-day intervention, 2-week washout)	Israel	39 subjects (33 females, six males)	Barley kernel-based bread (BKB) and white wheat bread (WWB)	Two groups of individuals were selected depending on their response (post-prandial glucose and insulin) to BKB	Bread: Stratification: Responders and non-responders in terms of blood glucose and insulin response to BKB Supplementation of the diet with WWB or BKB affected the microbial community in the responders but not in the non-responders. BKB ingestion compared to WWB ingestion resulted in significant changes in the relative abundance of specific members of the fecal microbiota in responders but not in non-responders After BKB consumption, the abundance of Bacteroidetes increased in the responders but not in the non-responders The *Prevotella*/*Bacteroides* ratio was higher after BKB compared with baseline in responders and after BKB in responders vs. non-responders, and *Prevotella copri* was the most abundant of the *Prevotellaceae* species in the responders. When transferred into mice, *Prevotella copri* improved glucose tolerance in mice fed a normal diet, but not in those fed a high-fat diet or a low-fiber diet
Li et al. (2024) (63) doi: 10.1002/mnfr.202400274	Glucose metabolism	Interventional, randomized trial, two groups	China	156 volunteers (one group consuming fermented rye bran, one group consuming refined wheat)	Fermented rye bran (FRB) and refined wheat (RW)	Responders or non-responders are identified according to whether blood glucose decreased by more than 10% after the rye intervention	Responders in FRB have a higher baseline Bacteroides count compared to non-responders (*P* < 0.001), which is associated with reduced blood glucose (*P* < 0.001), increased *Faecalibacterium* (*P* = 0.020) and Erysipelotrichaceae_UCG.003 (*P* = 0.022), as well as decreased 7β-hydroxysteroid dehydrogenase (*P* = 0.033) after intervention. Baseline Conjugated bile acids are marginally higher in responders of the FRB group. Tauroursodeoxycholic acid (TUDCA) and secondary bile acids decreased among responders in the RGB group
Aubin et al. (2024) (64) doi: 10.1111/jhn.13350	Metabolic flexibility (MetF) _ ability to switch between fat and glucose oxidation (use of respiratory quotient—RQ)	Interventional study Randomized—crossover (8 weeks intervention, 1–4 weeks washout)	France	39 subjects (22 females/17 males)	Control/multi-fiber bread	Two groups of individuals were selected depending on their MetF markers (delta RQ, mean RQ, RQ variance, fasting RQ, and Hill slope)	MetF responders (presenting a net change in delta RQ ≥ 0) exhibited higher baseline fasting low-density lipoprotein cholesterol (LDLc) levels and greater post-mixed-meal tolerance test triglyceride excursion (MMTT ΔTG)
Hadi et al. (2021) (65) doi: 10.1186/s12906-021-03351-w	Lipid profile and glycemic parameters	Systematic literature review of interventional studies	NA	Nine publications	Apple cider vinegar	No apple cider vinegar or cocoa product (placebo) or lower levels	Apple cider vinegar: after Stratification: a decrease in both triglycerides and total cholesterol concentrations was observed in studies conducted on type 2 diabetic patients, whereas those parameters were not decreased in non-diabetics A reduction of fasting plasma glucose is observed in non-diabetics following apple cider vinegar, but not in diabetics
Hadi et al. (2021) (65) doi: 10.1186/s12906-021-03351-w	Lipid profile and glycemic parameters	Systematic literature review of interventional studies	NA	Nine publications	Apple cider vinegar	No apple cider vinegar or cocoa product (placebo) or lower levels	Apple cider vinegar: after Stratification: a decrease in both triglycerides and total cholesterol concentrations was observed in studies conducted on type 2 diabetic patients, whereas those parameters were not decreased in non-diabetics A reduction of fasting plasma glucose is observed in non-diabetics following apple cider vinegar, but not in diabetics
Jafarnejad et al. (2020) (66) doi: 10.1007/s11906-019-1005-0	Blood pressure	Meta-analysis of interventional studies	NA	13 studies with 18 trials−758 participants	Cocoa	No cocoa or cocoa product (placebo) or lower levels	Cocoa: after stratification: there were differences in the efficacy of cocoa consumption on improving systolic and diastolic blood pressure and in subgroups of hypertensive subjects. No difference in the efficacy of cocoa was observed in normotensive patients and patients with elevated blood pressure
Ried et al. (2017) (51) doi: 10.1002%2F14651858.CD008893.pub3	Blood pressure	Meta-analysis of RCTs (35 trials): effect of chocolate or chocolate products on systolic and diastolic blood pressure in adults	NA	1,804 participants	Chocolate or chocolate products	No cocoa or cocoa product (placebo) or lower levels	Cocoa: after stratification: Significant systolic blood pressure-reducing effect of cocoa in the hypertensive subgroup, a trend toward blood pressure reduction in the prehypertensive subgroup, and a small non-significant effect in the normotensive subgroup
Petrone et al. (2013) (67) doi: 10.1007/s13668-013-0058-y	Endothelial function (FMD: flow-mediated dilatation)	Meta-analysis of RCTs (19 trials)	NA	454 participants (19 RCTs)	Dark chocolate or cocoa-containing beverages	No chocolate or chocolate product (placebo) or lower levels	Chocolate: when stratified for health status (healthy vs. one or more CVD risk factors), cocoa products or chocolate improve endothelial function more in the population with one or more CVD risk factors than in the healthy population
**Psychological disorders**							
Rintamäki et al. (2014) (31) doi: 10.3109/08039488.2013.851736	Depression, anxiety, and alcohol use disorders	Observational—cross-sectional	Finland	5,504 subjects (3,009 females and 2,495 males)	Dairy (cheese, fermented milk products) Cereals (rye and wheat), beverages (coffee and tea)	From a healthy population	Fermented milk product: decreased fermented milk product intake in females with alcohol use disorders (NS in males) Cheese: decreased cheese intake in females with anxiety disorders (NS in males) Tea: increased tea intake in males with anxiety disorders No conclusions on chocolate (particularly in males) when combined with sweets To be taken into account with sex: population characteristics: females suffering from anxiety disorders have a higher BMI, and males with anxiety disorders have a lower BMI than the remaining.
**Cancers**							
Kawakita et al. (2012) (68) doi: 10.1097/CEJ.0b013e32834f75b5	Upper erodigestive tract cancer risk	Observational—Case-control	Japan	3,836 subjects (959 patients with cancer, 2,877 sex-matched non-cancer control)	Yogurt	From no intake (to ≥1 time a day)	Yogurt: inverse association (whatever the quantity consumed above 0) with upper aerodigestive tract cancer risk (Not true for butter and milk) Decreased risk after stratification for BMI > 20

Focus on observational, interventional studies, meta-analyses, and systematic literature reviews.

#### 3.2.1 Impact of dairy, soy, and cocoa consumption on MetS and associated parameters

Several interventional studies reported variability associated with BMI and obesity. Pei et al. assessed the effects of low-fat yogurt consumption over 9 weeks in obese and non-obese individuals, examining biomarkers of chronic inflammation and gut permeability in both the fasted state (57) and fed state (58). Across these companion studies, lower tumor necrosis factor alpha (TNF-α) levels and TNF-α/sTNF-RII (TNF receptor II) ratios were observed in both obese and non-obese individuals following yogurt intake, indicating a reduced inflammatory status (57). This was accompanied by increased plasma IgM EndoCAb (immunoglobulin M endotoxin core antibodies) and decreased LBP/sCD14 (lipopolysaccharide-binding protein/soluble cluster of differentiation 14) levels, regardless of obesity status, suggesting a protective effect of this FF against chronic endotoxemia (57). These findings were further supported in the fed state by a lower incremental area under the curve (iAUC) for the LBP-to-sCD14 ratio and IL-6 concentration (58). However, differential responses to yogurt were also observed depending on the obesity status of volunteers. In the fasted state, yoghurt fed obese individuals exhibited lower diastolic pressure and higher plasma levels of 2-arachidonoylglycerol, which are markers indicative of improved intestinal barrier function, compared with obese controls. However, no similar effects were observed in normal-weight volunteers. In the fed state, pre-meal yogurt consumption resulted in a 72% higher net iAUC of sCD14 in the obese group (compared with consumption of a control snack), while non-obese individuals showed no significant response difference (treatment × obesity: *P* = 0.03). These results, although modest compared to the overall effects of yogurt on the measured parameters, suggest a more pronounced effect of yogurt in obese populations. Another interventional study investigated the effects of *Streptococcus thermophilus*-fermented milk in healthy or mildly hypercholesterolemic individuals with elevated LDL cholesterol level who consumed the product once daily for 12 weeks (60). Significant reductions in malondialdehyde-modified LDL (MDA-LDL), the MDA-LDL/LDL-cholesterol ratio, systolic blood pressure, and diastolic blood pressure were observed in participants receiving the *S*. *thermophilus*-fermented milk compared to controls. Notably, these reductions were statistically significant only among individuals with baseline MDA-LDL levels above the median, suggesting that yogurt may be more efficient in populations with already elevated MDA-LDL levels. The authors proposed that long-term suppression of oxidative stress by *S*. *thermophilus*-fermented milk could contribute to blood pressure reduction in individuals at a higher risk of cardiovascular disease, particularly those with elevated MDA-LDL levels. A similar differential beneficial effect of dairy was observed in an observational-longitudinal 6-year follow-up study, where overweight males with higher yogurt intake (highest quartile: 1.1–4.5 servings/day) showed lower weight gain and smaller increases in waist circumference compared to those consuming less yogurt (lowest quartile: 0–0.2 servings/day) (24). This association was not observed in normal-weight populations. Notably, similar results were also observed for milk consumption, suggesting that the fermentation of milk may not be the primary cause of the observed effect. Conversely, a different pattern emerged in a 7–10 year longitudinal study of perimenopausal females, where cheese consumption above one serving per day was associated with reduced weight gain among females with a BMI of ≥25 (56). Among females with a BMI of < 25, consumption of cheese, milk, or sour milk yielded the same effects.

Regarding the different responses of populations in lipid profiles and glycemic parameters, other FF were studied, particularly apple cider vinegar. Hadi et al. (65) conducted a systematic review and meta-analysis to assess the effects of apple cider vinegar on lipid profiles and glycemic parameters. The analysis revealed significant reductions in total cholesterol (TC), fasting plasma glucose, and HbA1c (glycated hemoglobin) concentrations. However, no significant effects were observed on low-density lipoprotein cholesterol (LDL-C), high-density lipoprotein cholesterol (HDL-C), fasting insulin, or the homeostatic model assessment for insulin resistance (HOMA-IR). Notably, as previously shown above with dairy products, the lipid-lowering effects were more pronounced in individuals with “non-healthy” or “metabolically disturbed” conditions, such as those with type 2 diabetes, as well as those undergoing interventions longer than 8 weeks. The effect of consumption of fermented soy was also evaluated in a 5-year follow-up observational longitudinal study with 7,552 volunteers on serum lipids. It showed that fermented soy and natto were capable of limiting the increase in cholesterol, non-HDL cholesterol, and LDL cholesterol in populations with a BMI of ≥25. In contrast, no significant effect was observed in populations with a BMI of < 25 (55).

Finally, the effects of cocoa, chocolate, and chocolate product consumption on blood pressure have been well studied in the literature, with three meta-analyses identified through our search (51, 66, 67). All three meta-analyses concluded an overall beneficial effect of cocoa on the reduction of blood pressure (with high heterogeneity between studies in the effect on systolic, diastolic blood pressure, or both). As summarized by Ried et al. (51), cocoa has a significant beneficial effect on reducing systolic blood pressure in the hypertensive population, with trends toward a similar but non-significant effect on prehypertensive and normotensive groups.

Our interpretation is that, as shown in nearly all the studies mentioned so far in this field, the beneficial effect of the FF studied (fermented dairy, cocoa, and apple cider vinegar) appears more pronounced in populations already presenting metabolic alterations or metabolic pathologies. Since the studies discussed above were interventional, one can argue that it may be more feasible to observe an improvement in a metabolic parameter or a biomarker that is out of its normal physiological range than to improve a parameter that is already at equilibrium and not supposed to be improved by any nutritional intervention.

#### 3.2.2 Impact of bread on insulin, post-prandial glycemic response, and lipid profile

Variable responses to carbohydrate-enriched foods have been reported in the literature, particularly following the ingestion of a frequently consumed fermented product, such as bread (see Table 4). Moazzami et al. (61) showed, in an interventional study, that whatever the bread tested (refined wheat vs. whole wheat bread), postprandial insulin response to the bread intake was higher in females, presenting higher blood concentrations of leucine and isoleucine and lower blood plasma sphingomyelins and phosphatidylcholines concentrations in the fasted state. These markers are associated with the early stages of insulin resistance development. This means that, in addition to the bread itself (which can contain more or less digestible carbohydrates), the metabolic profile of the study participants affects the insulin response. This was confirmed by Korem et al. (59) Kovatcheva-Datchary et al. (62), who demonstrated variations in blood glucose and insulin responses to white and/or whole breads that were not due to the different compositions of the breads. Indeed, Korem et al. (59) demonstrated that after 1 week of bread consumption (three meals of 50 g of available carbohydrate), high interpersonal variability was observed in the postprandial glucose response, which is attributed to the individual's fecal microbiome characteristics. This was also the case in the interventional study by Kovatcheva-Datchary et al. (62), who showed that microbial diversity in the gut could be modified by the kernel-based bread in some “responder” populations, whereas it was not or less the case in the “non-responder” population, with a *Prevotella*/*Bacteroides* ratio higher in “responders” than in “non-responders” after kernel-based bread intake. Another study by Li et al. (63) confirmed this concept in participants supplemented with fermented rye bran, where “responders” and “non-responders” groups were established based on their response in terms of a decrease in fasting blood glucose following 12 weeks of consuming rye bran bread. Participants presenting a decrease in fasting blood glucose of more than 10% were considered “responders.” The group of “responders” also presented a specific microbiota (higher baseline *Bacteroides*, increased *Faecalibacterium* and *Erysipelotrichaceae*_UCG.003) and altered secondary bile acids relative to “non-responders,” suggesting again a role of the individual gut microbiota (and its metabolism) in the variation of response of host glucose response to the fermented rye bran (and possibly the dietary fibers it contains). Variability of response to fiber-enriched breads (multi-fiber bread) has also been highlighted in another recent study (64), on metabolic flexibility assessed by respiratory quotient variation (as a proxy of the ability of metabolism to switch between fat and glucose oxidation) following a mixed-meal tolerance test. The variations in respiratory quotients before and after supplementation with the breads were not due to the nature of the bread itself but due to higher baseline fasting LDL-C and greater post-mixed-meal tolerance test triglyceride excursion (MMTT ΔTG). This suggests that the intervention was more beneficial to subjects presenting initial metabolic dysregulations. The analysis of the data does not allow us to evaluate the potential role of gut microbiota in the adaptations mentioned above, even if a companion study has shown an alteration of gut microbiota composition (and function) by the same multifiber bread (69).

In conclusion, bread appears to have been more extensively studied due to its potential to produce products with variable contents of starches and carbohydrates, presenting varying degrees of digestibility (i.e., more or less digestible fibers). However, the glycemic response to these breads depends more on the health status (and potential susceptibility to developing insulin resistance) and the microbiota diversity and composition of the host than on the fact that the food used to test glycemic response is fermented.

#### 3.2.3 Fewer studies outcomes: psychological disorders and cancer

Rintamäki et al. (31) investigated, in an observational cross-sectional study, whether cheese, tea, and fermented milk product intake could differ in populations suffering from depression, anxiety, or alcohol use. As individuals with anxiety disorders can also present increased or decreased BMI, the interaction between fermented dairy products, BMI, and the development of these psychological disorders should be studied in more detail. In the field of cancer, Kawakita et al. (68) found an inverse association between higher yogurt intake and a decreased risk of erodigestive tract cancer in an observational case-control study. This was not observed for butter and milk, suggesting a specific effect of yogurt. As for other outcomes presented earlier, the decreased risk was observed in populations with a BMI of >20, suggesting that the beneficial effect of yogurt to decrease the risk of digestive tract cancer is present in normal weight but not in lower weight populations (a population that may, due to its corpulence, consume less food, and yogurt).

### 3.3 Genetic background as a source of variability

Emerging evidence suggests that genetic differences may explain variability in individual health responses to FF. When genetic polymorphisms were considered as potential modifiers of FF effects, a diversity of studied FF and health outcomes was observed. However, one specific FF was studied much more extensively, namely coffee, for which genetic variants have been identified to explain variable effects on specific populations. This is the reason why factors affecting the response to coffee consumption have been presented separately from the other FF in this field, as summarized below.

#### 3.3.1 Coffee: a source of variability among populations, partly due to differences in their ability to metabolize coffee components

For tea and coffee, the final number of selected studies is 22 (19 meta-analyses, two systematic literature reviews, and one analysis combining three large cohorts). The majority of the meta-analyses and literature reviews selected in our search focused on the health effects of coffee but also addressed the question of variability of response (Table 5). The majority of the studies concerned cancer outcomes, including digestive, biliary tract, esophageal, renal, breast, hepatic, prostatic, ovarian, and lung cancers (50, 70–80). Other studies examined metabolic pathologies (MetS, Metabolic Dysfunction Associated Steatotic Liver Disease or MASLD, and hypertension) (85–89), urinary disorders (81, 82), hip fracture risk (91), and psychological disorders (90). A few studies have focused on the impact of coffee on functions such as inflammatory responses and pathologies, as well as C-reactive protein (CRP) levels specifically (83, 84).

**Table 5:** Coffee and/or tea intake and analysis of the variability of response within populations in various physio/pathological situations: cancers, urinary tract disorders, inflammation and inflammatory pathologies, MetS-chronic metabolic pathologies, and psychological disorders.

**Reference**	**Specific outcome measured**	**Study design**	**Number of studies Type of studies**	**Variability of response observed in the outcomes measured**
**Cancers**				
Xie et al. (2016) (70) doi: 10.6133/apjcn.092015.07	Risk of gastric cancer	Meta-analysis—Observational studies	22 studies (nine cohort, 13 case-control)	Hospital-based control studies:
				decreased risk with coffee intake (RR (relative risk) (95%CI): 0.75 (0.66–0.86). This is not the case in population-based or cohort studies Population: Europe: decreased risk with coffee intake 0.86 (0.75–0.98). No effect of coffee on the risk in Asian or other populations Beneficial effects of coffee when studies were conducted after 2004: 0.71 (0.51–0.99) vs. studies conducted between 1,994 and 2,003 No effect of coffee intake on risk when stratified by biological sex
Zhang et al. (2018) (71) doi: 10.1097/MD.0000000000010514	Esophageal cancer incidence	Meta-analysis—Observational studies	11 studies: case-control and cohort studies	No significant effect of coffee (highest intake vs. lowest/no intake) on esophageal cancer risk RR (95%CI), 0.91 (0.73–1.14) When stratified by sex and type of epidemiologic study, no association was found between coffee consumption and esophageal cancer incidence The beneficial effects of coffee in the East Asian population are 0.64 (0.44–0.83), which is not observed in the Euro-American population, 1.05 (0.81–1.29)
Wijarnpreecha et al. (2017) (72) doi: 10.1111/imj.13621	Risk of renal cell carcinoma (RCC)	Meta-analysis—Observational studies	22 studies, 16 case-control, 6 cohort studies	Coffee is not a risk factor for RCC No effect of coffee intake on risk when stratification by biological sex
Tang et al. (2009) (73) doi: 10.1016/j.ajog.2008.10.019	Risk of breast cancer	Meta-analysis—Observational studies	18 studies, nine case-control, and nine cohort studies	A slight borderline beneficial effect of coffee was considered by authors in the population in the USA (RR (95%CI): 0.94 (0.87–1.01), not in Europe 0.95 (0.86–1.06), and even less in Asia 0.92 (0.64–1.33)
Lafranconi et al. (2018) (50) doi: 10.3390/nu10020112	Risk of post-menopausal breast cancer	Meta-analysis—Observational studies	21 prospective cohort studies	Borderline global beneficial effects of coffee on overall populations RR (95%CI): 0.96 (0.93, 1.00) Beneficial effect in post-menopausal females (0.92 (0.88, 0.98), not premenopausal 0.98 (0.89, 1.07) No significant increased/decreased risk according to geographical location (even if considered as borderline beneficial by authors for North America), estrogen/progesterone receptor status, coffee type (decaffeinated or not), BMI, duration of follow-up, smoking status, alcohol intake, physical activity, and education
Li et al. (2013) (74) doi: 10.1371/journal.pone.0052681	Risk of breast cancer	Meta-analysis—Observational studies	16 studies: six cohort and 10 case–control	Borderline global beneficial effect of highest coffee consumption on overall populations RR (95%CI): 0.96 (0.93, 1.00) Beneficial effects of coffee consumption on Estrogen Receptor (ER) status negative population [0.81 (0.67–0.97)]. Not true for ER positive [1.01 (0.93–1.09)] No significant increased/decreased risk according to geographical location (even if more borderline in North America, 0.97 (0.92–1.01), and Europe: 0.96 (0.90–1.02) than Asia, 0.92 (0.64–1.33), pre-post-menopausal status, type of studies (cohort vs. case-control)
Godos et al. (2017) (75) doi: 10.3390/nu9090950	Risk of Biliary Tract Cancers and Liver Cancer	Meta-analysis—Observational studies	18 case-control and prospective cohort studies	Beneficial effects of coffee (highest intake vs. lowest intake) to limit risk of liver cancer in both case-control populations, RR (95%CI) 0.48 (0.33, 0.70), and prospective cohort studies: 0.53 (0.41, 0.69) Beneficial effect in males and females (whatever the type of study: case-control or prospective) Beneficial effect in North America 0.72 (0.52, 0.98), Europe, 0.48 (0.36, 0.64), and Asia, 0.42 (0.30, 0.58) Beneficial effect of caffeinated coffee 0.65 (0.49, 0.86), not decaffeinated, 0.85 (0.63, 1.14). Beneficial effects in smokers and non-smokers and in the population with or without chronic hepatitis
Yu et al. (2016) (76) doi: 10.1038/srep37488	Liver cancer risk	Meta-analysis—Observational studies	10 prospective studies	Beneficial effects of coffee (highest intake vs. lowest intake) to limit the risk of liver cancer (RR). True for males and females, whatever the study regions (Asia, Europe, or North America), with or without a history of disease, regardless of the BMI or education level
Larsson and Wolk (2007) (77) doi: 10.1053/j.gastro.2007.03.044	Liver cancer risk	Meta-analysis—Observational studies	12 case-control and nine cohort studies	An increase in consumption of 2 cups of coffee per day was associated with a 43% reduced risk of liver cancer RR (95%CI), 0.57 (0.49–0.67). Remain true whether or not the populations had a history of liver diseases
Lu et al. (2014) (78) doi: 10.1007/s10552-014-0364-8	Prostate cancer risk	Meta-analysis—Observational studies	12 case-control and nine cohort studies	Beneficial effects of coffee (highest intake vs. lowest intake) to limit risk of liver cancer ORs (Odds Ratios) (95%CI) 0.91 (0.86, 0.97). Beneficial effect in cohort studies (0.89 (0.84–0.95), not in case-control 1.10 (0.95–1.26). Beneficial effect in the USA 0.93 (0.87–0.99) and Europe 0.87 (0.78–0.97), not Canada 1.05 (0.81–1.28) or Asia 0.76 (0.48–1.04). Beneficial effect recorded in studies of high quality 0.91 (0.85–0.97), not low quality 0.93 (0.82–1.04)
Tang et al. (2010) (79) doi: 10.1016/j.lungcan.2009.03.012	Lung cancer risk	Meta-analysis—Observational studies	Five cohort prospective and eight case-control studies	Detrimental effect of coffee (highest intake vs. lowest/no intake) on lung cancer risk RR (95%CI), 1.27 (1.04–1.54) Remain detrimental for studies localized in America 1.33 (1.07–1.65), Japan 1.34 (1.05–1.70), but not Europe 1.26 (0.76–2.09) Non-significant effect in smokers, 1.28 (0.87–1.88), and borderline beneficial effects of coffee in non-smokers, 0.78 (0.60–1.00) Two studies on decaffeinated coffee show a beneficial effect 0.66 (0.54–0.81)
Berretta et al. (2018) (80) doi: 10.18632/oncotarget.24829	Ovarian cancer risk	Meta-analysis—Observational studies	Eight prospective cohort studies	No association between ovarian cancer and coffee intake No association after stratification for geographical area, menopausal status, coffee type (decaffeinated or not), BMI, education, and alcohol intake
**Urinary tract disorders**				
Wang et al. (2014) (81) doi: 10.1159/000356559	Urolithiasis risk	Meta-analysis—Observational studies	Four case-control and three cohort studies	Beneficial effects of coffee (highest coffee consumption) on urolithiasis risk OR (95%CI), 0.70 (0.60–0.82) Remain beneficial when stratified for study design (cohort vs. case-control) and biological sex. Beneficial in the United States 0.64 (0.55–0.75) (5 studies) but not significant in Europe 0.71 (0.49–1.02) (1 study) or Asia 0.94 (0.67–1.32) (1 study)
Sun et al. (2016) (82) doi: 10.1186/s12894-016-0178-y	Risk of urinary incontinence	Meta-analysis—Observational studies	One case-control, two cohort, and four cross-sectional	No association between coffee intake (regular vs. non-consumption of coffee/caffeine) and risk of urinary incontinence for the overall population OR (95%CI), 0.99 (0.83–1.18) Remain non-significant when stratified by biological sex
**Inflammation/inflammatory pathologies**				
Moua et al. (2020) (83) doi: 10.3390/nu12051349	C-reactive protein (CRP) in blood	Meta-analysis—Observational studies	10 cohort studies	No association between the increase in coffee intake and CRP blood levels in the overall population No further association when stratified by biological sex and geographic location However, three studies, with the largest sample size, observed a statistically significant association between coffee and CRP levels, which was inverse among European and United States (US) females and Japanese males (1.3–5.5% decrease in CRP per 100 ml of coffee consumed) and positive among European males (2.2% increase)
Lee et al. (2014) (84) doi: 10.1007/s10067-014-2631-1	Risk of rheumatoid arthritis	Meta-analysis—Observational studies	Two case-control studies and two cohort studies	Trend of a positive association between total coffee intake and RA incidence (detrimental effect) (relative risk [RR] of the highest vs. the lowest group=4.148, 95% confidence interval [CI]=0.792–21.73, *P* = 0.092). This was also the case for the two case-control studies, but not for the cohort studies. When stratified by coffee type intake, the association is no longer significant for caffeinated or decaffeinated coffee. Important variabilities between studies
**Metabolic syndrome**				
Virgili et al. (2023) (85) doi: 10.1111/fd12606	Cardiometabolic outcomes	Systematic literature review	17 studies (four RCTs, one interventional and quasi-experimental study, three case-control studies, three cross-sectional studies, six cohort studies)	Caffeine can increase glucose levels by different mechanisms. Single-nucleotide polymorphisms (SNPs) of CYP1A2 rs762551 and ADORA rs5751876 genes were associated with glucose response when caffeine was consumed with carbohydrates. Caffeine is widely recognized as a potent inducer of an overall acute hypertensive response. CYP1A2 rs762551 moderated the association between coffee intake and hypertension. ADORA2A rs5751876 and the ADRA2B I variants moderated the associations between caffeine and blood pressure. Studies that investigated the effects of genetic variations on CVD and caffeine consumption reported equivocal findings (CYP1A2) or warrant replication (COMT, ADORA, and TRIB1). As a consequence, consumption of adequate amounts of coffee should not be discouraged. However, because some of the variants cited above are involved in caffeine metabolism, the healthy eating recommendations for coffee should be adapted (tailored) to the genetic profiles of each individual. This is because current maximal recommendations may not be safe enough for slow metabolizers to limit the occurrence of hypertension, pre-diabetes, or heart attacks
Wong et al. (2021) (86) doi: 10.1093/advances/nmaa132	Metabolic syndrome (MetS) risk	Meta-analysis—Observational studies	15 studies. 13 cross-sectional studies, two longitudinal	No association between coffee intake (highest consumption) and MetS for all populations, even if adjusted for sex However, when stratification is made, high intake of coffee is beneficial to limit MetS in males OR (95%CI), 0.78 (0.71–0.93), and in females, 0.72 (0.65–0.79) Additionally, only moderate coffee consumption in Caucasians, but not Asians, was associated with lower odds of having metabolic syndrome The difference between the two results (sex-adjusted and sex-stratified) can come from the different types of studies used in these two analyses (the inclusion of a new study with 130,420 participants that observed a significant inverse association between coffee consumption and MetS in both males and females)
Haghighatdoost et al. (2023) (87) doi: 10.3390/nu15133060	Hypertension risk	Meta-analysis—Observational studies	25 studies (13 cross-sectional studies, 12 cohort studies	Prospective cohort studies: beneficial effect of the highest coffee consumption to limit hypertension risk RR (95%CI), 0.93 (0.88–0.97). This is also true for cross-sectional studies OR 0.79 (0.72–0.87) In cohort studies, the beneficial effect of coffee in the US was 0.92 (0.87–0.97), but not in Europe, 0.97 (0.83–1.13), or Asia, 0.94 (0.83–1.07). Significant effect also when cases were >3,000, 0.93 (0.89–0.97), whereas it is not true for cases < 3,000, 0.94 (0.84–1.06). Beneficial in females (4 studies) 0.93 (0.88–0.98), not males (1 study) 1.07 (0.67–1.70). Moreover, it depends on the blood pressure at baseline In cross-sectional studies, beneficial effects were observed in Europe, 0.72 (0.62–0.84), and in Asia, 0.87 (0.81–0.95) (no data for the US). The effect was beneficial in males (4 studies), 0.75 (0.61–0.93), but not females (two studies), 0.88 (0.73–1.06), and also depended on blood pressure at baseline
Henn et al. (2023) (88) doi: 10.1016/j.ajcnut.2023.09.023	Long-term weight gain	Observational study—Analysis of three large US cohort studies	Three prospective cohort studies (NHS, NHSII, HPFS, and cohorts in the United States of America)	Each one-cup-per-day increment in unsweetened caffeinated coffee was associated with a reduction in 4-year weight gain of −0.12 kg (95% CI: −0.18, −0.05 kg) and of −0.12 kg (95% CI: −0.16, −0.08 kg) for unsweetened decaffeinated coffee. True for females and males. Adding sugar is associated with weight gain Regarding stratification: Stronger magnitudes of the observed associations were observed at younger age ( ≤ 50 y.o: −0.16 kg, 95% CI: −0.22, −0.10; >50 y.o.: −0.06 kg, (−0.08, −0.04) and a higher baseline of BMI (BMI < 25: −0.07 kg (−0.11, −0.02); BMI ≥30: −0.24 (−0.30, −0.18)
Nemati et al. (2024) (89) doi: 10.4103/abr.abr_174_24	General obesity and abdominal obesity risk	Meta-analysis Cross-sectional studies	23 studies (15 studies on abdominal obesity, 12 studies on general obesity)	General obesity: Coffee consumption increased the risk of general obesity in females (OR: 1.84, 95% CI (1.51, 2.24), not in males, 0.99 (0.80, 1.23), nor in the overall population, 1.05 (0.99, 1.12) No effect of coffee in Europe 1.03 (0.96, 1.11), but increased risk in Asia 1.18 (1.08, 1.28) When FFQ is used: increased risk: 1.48 (1.32, 1.66). Not true when FFQ is not used: 1.00 (0.93, 1.06) Abdominal obesity: No effect of coffee in both males and females Decreased risk both in Europe 0.90 (0.83, 0.96) and Asia 0.89 (0.85, 0.93) Decreased risk when sample size is >1,000: 0.89 (0.86, 0.92). Not true when < 100: 1.15 (0.88, 1.49) Decreased risk when FFQs are not used: 1.15 (0.88, 1.49), not true when FFQs are used: 0.97 (0.92, 1.02)
**Psychological disorders**				
Kapellou et al. (2023) (90) doi: 10.1093/nutrit/nuad029	Brain-related outcomes: cognitive performance, anxiety, sleep disturbance/insomnia	Systematic literature review: observational/interventional studies	22 studies (15 randomized controlled trials, six cross-sectional studies, one genome-wide association study)	Variability in the CYP1A2 and the ADORA2A genes is associated with brain-related outcomes of caffeine. Nevertheless, it is not yet clear what specific genotypes are implicated in each brain outcome, which functions of cognition are particularly associated with caffeine (simple vs. complex), whether there are gender differences in the effects of caffeine on anxiety, and how habitual caffeine intake may influence the acute effects of caffeine. The variability, in addition, genes may be involved in caffeine pharmacokinetics and brain neurotransmission, collectively influencing individual responses to caffeine

Focus on meta-analyses, systematic literature reviews, and studies combining large cohorts.

Based on the results of these meta-analyses, it is challenging to draw unequivocal conclusions about the significant beneficial or detrimental effects of coffee intake (or increased intake) on the outcomes listed above. This is due either to the high variability between studies or to the fact that different meta-analyses examining similar outcomes arrived at inconsistent conclusions. For instance, in the case of cancer, as summarized by Pauwels and Volterrani (92), if coffee intake is associated with a lower risk of all-cause mortality [as also described by Poole et al. (93)], the picture becomes less clear when it comes to specific types of cancer.

The authors highlight important uncertainties both between and within studies that can explain the difficulty in drawing definitive conclusions. Consequently, because the high variability in responses within studies could mask potentially significant correlations, the authors conducted additional *a posteriori* analyses to further explore possible mechanisms of action. These analyses considered several sources of variability by (i) analyzing certain studies separately (e.g., based on study design or location of the population studied), (ii) stratifying data within studies according to specific population characteristics (e.g., biological sex and smoking status), and (iii) including these sources of variability as covariates in statistical models. This stratified analysis revealed that, for some cancer risks, the location of the study (Europe, America, or Asia), as well as biological sex and hormonal status in hormone-dependent cancers, may lead to different outcomes. For instance, while increased coffee intake is not clearly correlated with a higher/lower risk of breast cancer, a negative correlation has been observed in estrogen receptor-negative populations [though not confirmed by Lafranconi et al. (50)], European and American (but not Asian) populations [see (74), also not confirmed by Lafranconi et al. (50)], and in post-menopausal females (50). In conclusion, stratification can help reveal significant correlations; however, this approach reduces the number of studies included in the meta-analysis and, consequently, the power and reliability of the conclusions (as shown in the example cited above, where inconsistent results were obtained after stratification in meta-analyses conducted on breast cancer). This limitation is clearly highlighted in the study by Wong et al. (86), where the conclusion regarding the effect of coffee on MetS differed depending on analytical approach: no effect was observed when data were adjusted for biological sex, whereas significant beneficial effects of coffee were found for both males and females when stratification was applied. Therefore, the analytical method, the selection of studies, and the relative importance of some studies with large sample sizes can skew (or significantly impact) the significance and/or variability of the final results.

In the concluding remarks of the meta-analyses on the health effects of coffee intake, the authors emphasize the difficulty of accounting for all confounding factors. For cancer, even if some constituents of coffee are suspected to carry some antiproliferative, antioxidant, and anticancer properties [e.g., caffeine, polyphenols, diterpenes (cafestol and kahweol), and chlorogenic acids] (92), too many confounding factors are not taken into account, sometimes intertwined and not detailed in observational studies. These sources of variability are population characteristics (biological sex, age, physical activity, education level, body weight, abdominal obesity, genotype [e.g., CYP1A2 variants, estrogen receptors + or -, hormonal status (pre- or post-menopause)], location of studies (Europe, USA, Asia; more or less associated with ethnicity), study design included in the analysis (case-control and/or cohort and/or interventional study), sample size, utilization of food frequency questionnaires (FFQ) for evaluation of consumption only [quantity—generally the notion of cup is used, habits of coffee consumption (e.g., with or without milk/sugar)], lack of precision in coffee-making process [type of coffee used, brewing process, temperature of coffee ingested (and impact on esophageal cancer for instance)], life habits that could be correlated with coffee intake (e.g., diet, smoking, alcohol consumption), the type of disease (e.g., for a same organ, type of cancer), design of studies. If some of the variables can be included in models as confounding factors (e.g., biological sex and body weight), others are not detailed in the protocols (e.g., quantity of coffee, coffee-making process, and details on diet). All these parameters impact the quantity of available molecules present in coffee capable of driving the healthy/non-healthy effect. The description of the coffee intake is also closely intertwined with the geographical location of studies, as the composition and quantity of a cup of coffee vary between Europe, America, and Asia due to population-specific consumption habits. It should be noted that data are lacking for other populations with coffee consumption (Africa, South America). This issue concerning confounding factors applies to all FF studied above but is particularly evident in the case of coffee, possibly due to the importance of the factors involved or because of the larger number of studies that allowed for a more extensive analysis of these factors.

Either way, all authors agreed on the necessity of increasing the number of studies and data to minimize biases and facilitate a proper evaluation of the risks and benefits associated with coffee in various health outcomes mentioned above. However, when looking at the wide sources of variability, particularly when it comes to coffee intake, and because the majority of the evaluations are made using data from observational studies, a detailed evaluation/standardization of the notion of a “cup of coffee,” combined with information on the sources of beans and the processes of grinding, fermentation, and brewing, are needed.

Finally, we noticed that one of the fields of research where interventional studies have been carried out extensively on the impact of coffee on health and variability of response evaluation is the genetic capacity to metabolize caffeine, which is closely associated with some clearly identified genetic variants. As caffeine is hypothesized to have an impact on health on both an acute and long-term basis, variants in populations have been more thoroughly investigated. To explain important variabilities of responses observed in populations in terms of mortality risk, metabolic pathologies (T2D and cardiovascular), cancer, or psychological disorders to coffee intake, genome-wide association studies have been carried out, and genetic variants were found (near Cholesterol 7α-hydroxylase—CYP1A1/2 genes, Aryl Hydrocarbon Receptor—AHR genes, and Adenosine 2A Receptor [ADORA2A] gene). Specific alleles of these genes were associated with higher or lower coffee consumption, reflecting the differences in metabolic capacity or susceptibility to caffeine. This is caused by the capacity of these populations to metabolize coffee, as these genes (and others) are involved in caffeine metabolism and/or sensitivity/susceptibility to coffee/caffeine [for details, see (82, 87, 94)]. Other genetic variants have also been discovered in the field, but they have been studied or are being studied more recently.

#### 3.3.2 Other genetic variants identified in variable response to FF other than coffee

Among the three observational studies identified in our search, health outcomes associated with yogurt, fermented milk, and bread differed according to genetic variants. Karami et al. (95) reported a genotype-specific association between yogurt consumption and renal cell carcinoma (RCC), observing an increased risk of RCC with higher yogurt intake among individuals with the wild-type CC genotype at single-nucleotide polymorphisms (SNPs) rs3118538 and rs10776909 in the *Retinoid X Receptor Alpha* (*RXRA*) gene. Zhang et al. (96) demonstrated variation in cardiovascular (CVD) outcomes based on lactase persistence genotype (rs4988235), wherein participants with high fermented milk intake, CVD, and CVD mortality risk were significantly higher in lactase-persistent individuals (CT/TT). Additionally, Westerman et al. (97) demonstrated variability in glycemic control response (HbA1c) to bread consumption modulated by the *Transient Receptor Potential Cation Channel, Subfamily M, Member 2* (*TRPM2*) rs62218803 polymorphism, where alternate allele carriers experienced a significantly reduced effect compared to reference homozygotes.

Three intervention studies similarly highlighted genetic variability in responses to FF, including doenjang (Korean fermented soy paste) and cheese. Lee et al. (98) showed that 12-week supplementation with doenjang improved visceral fat catabolism, specifically in individuals carrying the mutant G allele of *uncoupling protein-1* (*UCP-1*), which was potentially mediated by increases in free fatty acids and insulin. Similarly, Cha et al. (99) found that doenjang supplementation led to a significant reduction in visceral fat area, more pronounced among individuals with the mutant T allele of the *peroxisome proliferator-activated receptor* (*PPAR-*γ*2*) gene compared to wild-type carriers. Antioxidative responses (measured by oxygen radical absorbance capacity and catalase activity) also differed by genotype and were increased in wild-type C allele carriers. Rajendiran et al. (100) identified variability in triglyceride levels following cheese intake, linked to SNPs (NPC1L1-rs2073547, PPAR-rs6008259) and *apolipoprotein E* (*APOE*) isoforms, which affect both the magnitude and direction of the lipid response.

Collectively, these findings highlight genetic polymorphisms in genes such as *PPAR-*γ*2, APOE, TRPM2*, and *RXRA*, as well as those related to lactase persistence, as important modulators of cardiovascular, metabolic, and cancer-related outcomes or parameters linked positively or negatively to FF intake. Future studies should investigate the clinical relevance of these genetic variations and their impact on health responses to inform personalized dietary recommendations more effectively. An ongoing consolidation of data on genetic variants, specific FF types, intake patterns, and health outcomes is also necessary. Public health strategies could eventually integrate knowledge of the prevalence of genetic variants of interest, the clinical significance of responses, and the strength of evidence to prioritize dietary guidelines based on genetic predisposition.

### 3.4 Other sources of variability

#### 3.4.1 Gut microbiota as an increasingly recognized source of variability

The gut microbiota is a dynamic, spatiotemporal interface between diet and host health, facilitating the metabolism or transformation of dietary components into end-products of fermentation, such as short-chain fatty acids (SCFAs) (101). It plays a critical role in health and has been identified as altered in cardiometabolic diseases, cancer, and psychological disorders (102). There are significant interindividual differences in gut microbiota composition and function, with the subject accounting for the major source of variation, up to 70%, in composition (103). Host and environmental factors, such as sex, genetics, age, or geographical origins, contribute to the variable part (104). Recent studies have highlighted the contribution of gut physiology and environment (e.g., transit time, pH) to variations in gut microbiota (105). Diet is among the most extensively studied environmental factors influencing the gut microbiota, with the entire diet contributing the most to variation (106). All these variables may affect the gut microbiota, which, in turn, could differentially interact with the host and/or confound results in the context of comparative analysis.

Among diet components, FF items may contribute to overall (even if small) variation (107). To date, few studies have specifically investigated the association of FF, including yogurt and coffee, with the gut microbiome and health outcomes in large-scale studies, primarily in Western populations (108–110). However, no studies have examined factors associated with variation in this association. In addition, these studies differed in covariate adjustment, with some adjusting for overall diet quality to specifically disentangle the contribution of specific FF to microbiota variation beyond overall healthier dietary habits (108). However, studies on how variation in gut microbiota can explain differential responses to FF consumption are limited, partly due to a lack of power to stratify both the microbiome and the response to FF in intervention studies. More specifically, in this section, we will describe how FF consumption can affect the gut microbiota composition and function, leading to differential effects on health outcomes, and how variable responses to FF consumption can depend on the initial microbiota function and composition.

#### 3.4.2 Ingestion of live microbes

Overall, the consumption of fermented products containing live microbes consistently results in a transient increase of fermented-food taxa in the gut (108, 109, 111), with a dose-response effect. This has been mostly studied in the field of fermented dairy products (112) and rarely in plant-based fermented foods (113). The viability of microbial composition in these foods depends on factors such as pH, water activity, and nutrient composition, which influence their potential to colonize the gut upon ingestion and interact with resident communities (114). Recently, a large observational study (NHANES 2001–2018) reported a positive association between dietary intakes of live microbes and a variety of health outcomes, including lower systolic blood pressure, lower plasma triglycerides, and a lower BMI (115). Since many FF contain high concentrations of lactic acid bacteria (10^5^-10^9^ UFC/ml), they are an important source of live microbes, which could then promote a better health status (1). Besides live microbes, compounds produced in the product via fermentation processes may have a direct or indirect (through action on the host gut microbiome) effect on host parameters.

The gut microbiome of individuals plays a role in responses to fermented foods, leading to the concept of clinical “responders” and “non-responders.” For example, the consumption of a 5-strain fermented milk product for 28 days led to an improvement in gas-related symptoms in some people (“responders”) and not in others (“non-responders”) (116). In “responders,” fermented milk intake induced an increased abundance of *Faecalibacterium prausnitzii*, changes in bacterial motility and lysine degradation pathways, as well as enhanced methanogenesis, reducing intestinal gas volume. These findings suggest that *F. prausnitzii* may reduce gas by shifting microbial activities, promoting methane production, and stimulating the growth of beneficial bacteria. *F. prausnitzii* also alleviated symptoms induced by a flatulogenic diet, with bacterial species activating CAZymes for carbohydrate breakdown, likely metabolizing an excess of carbohydrates. The response difference between groups was attributed to baseline microbiota composition, with “responders” having a different initial microbiota profile (116).

One example of the effect of FF consumption on the gut microbiota and health differential response was indirectly shown by the measurement of plasma soluble CD14 as a biomarker of gut barrier function in a cross-sectional study from the two US cohorts, the Nurses' Health Study (NHS) and the Health Professionals Follow-up Study (HPFS) (40). Higher yogurt consumption (at least two cups per week) was inversely associated with plasma concentrations of soluble CD14 in males, but not in females; however, the mechanism is unknown. The absence of effect in females was attributed to the fact that females's fecal microbiota had a higher fecal pH, as well as a higher abundance of total bacteria, *Bifidobacterium*, and the *Lactobacillus gasseri* subgroup, possibly limiting the effect of ingested bacteria from yogurts due to a lower available metabolic niche.

While the majority of studies have focused on the fecal microbiome, a growing number of studies examine the effect of probiotic and fermented milk products on the small intestine microbiota, an intestinal location with poorly characterized host-microbiota interactions. Especially, lactic acid bacteria are metabolically well-suited for the small intestine environment, with the ability to metabolize simple carbohydrates (117). One study explored the activity and effect of two fermented milk products (yogurt containing starter culture bacteria *Streptococcus thermophilus* and *Lactobacillus delbrueckii* subsp. *bulgaricus*) and one containing a strain of *L. rhamnosus* (~10^11^ CFU/day) on the ileal microbiome in patients undergoing ileostomy. The most abundant strains of both products (*L. rhamnosus* and *Streptococcus thermophilus)* were transiently detected (8.5 and 5.2% of the total microbiota, respectively, with relative abundances of up to 90%), exhibiting high intra- and inter-subject variability. Microbial taxa such as *Peptostreptococcaceae* were positively associated with a low abundance of ingested bacteria, possibly due to competition in the small intestine (118). Other microbiota located outside the gut can also be influenced by FF intake, as shown in an observational study involving 600 females, which reported a positive correlation between dairy consumption and vaginal *Lactobacillus* profile, potentially related to the risk of preterm birth (47). Thus, analyzing microbial compartments other than feces could lead to the identification of additional between-subject variation, which may be targeted with personalized nutrition approaches.

#### 3.4.3 Other components in the FF

In addition to a direct effect of live microbe ingestion, the presence of nutrients and compounds within FF could be responsible for the overall FF effect, as well as the potential role of these molecules in the variability of the host's response to FF. There are very few studies on the potential specific role of the fermentation process (or the presence of specific molecules or microbial components in the product) on inter-subject microbiota variations. As explained above, individual variation in the host microbiota may also be responsible for a lack of effect of the nutritional intervention. Indeed, dietary acculturation to the Western diet among migrants is accompanied by a loss in gut microbiome diversity and function, leading to an inability to metabolize fibers, thus predisposing individuals to metabolic diseases (119). Consequently, gut-microbiota-targeted diets are suggested to compensate for this loss by enriching microorganisms with fiber-degrading activity, along with dietary fibers in the diet (120). Currently, it is unknown if fiber-rich FF containing live microbes capable of degrading dietary fibers could enrich and complement gut microbiota functionality.

The pioneering work by Zeevi et al. (121) aimed to assess the influence of food intake on gut microbiota and the impact of gut microbiota on glycemic responses. The authors demonstrated in a cohort of 800 individuals that the postprandial glucose response varied significantly among individuals and that gut microbiome composition was a significant explanatory factor. They developed an algorithm capable of identifying multiple metabolic parameters, dietary habits, physical activity, and bacterial taxa as either beneficial or non-beneficial. They were able to predict the postprandial glucose response to personalized dietary intervention. This protocol is among the first steps toward individualized nutritional recommendations, but it was not designed to address the specific effect of FF supplementation efficiency, even if the diets contained some FF (121). In another study, glycemic response to bread could be predicted from the baseline microbiome (59). The importance of microbial enterotype composition and function in the differential response to whole grain products with fermented rye bran, compared with refined grain, was also demonstrated in a study of Chinese adults (79 participants, 12 weeks of consumption). Suggested mechanisms included alterations in SCFA and bile acid metabolism as potential mediators of the observed beneficial effect of whole grain bread on glucose metabolism (63).

#### 3.4.4 Diet effects—Including FF

Some data are also available on dietary patterns rather than the specific effects of FF on different health outcomes. A recent study examined whether dietary patterns related to MetS differ according to gut microbial enterotypes among 348 Korean adults aged 18–60 years, recruited between 2018 and 2021, in a cross-sectional study. The authors suggested that dietary factors associated with MetS risk may differ based on the gut microbiomes of Korean adults. More precisely, the *Bacteroides* enterotype, mainly found in people eating more refined rice-based diets, and the *Prevotella* enterotype, more frequent in people with a low fermented food-based diet, were associated with an increased risk of MetS (122).

Links between a pathology, gut microbiota composition and function, and diet, including FF, can be highlighted in observational studies; however, causality links are not always possible to assess due to the study design. Indeed, in the study from Baragetti et al. (123), the authors showed associations between diet, changes in taxonomic gut microbiome, and subclinical carotid atherosclerosis (SCA), with subjects without SCA reported to consume a higher amount of cereals (*P* = 0.009), starchy vegetables (*P* = 0.027), dairy products and beverages (*P* = 0.004 and *P* = 0.016, respectively), yogurts (*P* = 0.047), and bakery products (*P* < 0.001) as compared to those with SCA. These dietary intakes were more closely correlated with bacterial genera in subjects with SCA compared to those without SCA. Data supported the interaction between dietary exposure and changes in gut microbiota at the early stages of SCA. A reduced contribution of pathways (such as starch degradation and sulfur oxidation, and the biosynthetic routes of purine and pyrimidines) encoded by *F. prausnitzii* was found in metagenomes of +Intima-Media Thickness (IMT)/+SCA subjects. Notably, *F. prausnitzii* has been previously reported to be actively involved in gut permeability through the production of the anti-inflammatory compound butyrate. To address causality, an increasing number of interventional studies specifically dedicated to FF consumption were recently implemented. Wastyk et al. (124) conducted an FF-enriched nutritional intervention (36 participants, 27 weeks in duration) where the quantity and diversity of FF were increased based on participants' preferences, including yogurt, kefir, fermented cottage cheese, kombucha, vegetable brine drinks, and fermented vegetables such as kimchi. A positive correlation was observed between the total number of servings and microbial diversity. Yogurts and vegetable brine drinks were the most commonly consumed FF, and a stronger correlation was observed with these types of FF. The authors suggest that FF consumption was not primarily due to the consumption of microbes but rather indirectly affected microbiota diversity by increasing the representation of strains present but below the detection level. FF intake was also associated with a decrease in markers of host inflammation. Despite significant advances and the fact that each individual served as their own control (interventional study), the study lacked a control arm (on a usual diet) or comparative data using non-fermented, corresponding products.

The distinction between the effect of fermentation and the ingestion of microorganisms together with the FF was illustrated recently in a randomized controlled trial comparing pasteurized vs. fresh sauerkraut using a crossover design (87 participants, 4-week consumption) (113). Despite the global absence of effects on the microbial profile in the two intervention periods, some specific outcomes were related to interpersonal variations. For example, significant variations in the relative abundance of species from the *Lachnospiraceae* family were observed among overweight or older participants. The changes in microbial diversity were also consistently smaller for participants with a higher baseline diversity. As expected, the main bacterium found in sauerkraut (*Lacticaseibacillus paracasei*) was found in fecal samples after consumption of fresh sauerkraut. Future studies comparing FF to different controls (non-fermented, fermented, and pasteurized) are needed to precisely assess the effects of fermentation (bioactive compound production) and possibly disentangle the effect of live microbes from dead strains.

#### 3.4.5 Individual variability in responses to fermented foods: the “responder” vs. “non-responder” concept

The concept of “responders” and “non-responders” has also been demonstrated for parameters other than gut microbiota. In a randomized controlled study, the glycemic index of eight experimental food products, including two types of bread, a cake, a cookie, and a fruit drink, was assessed in 10 volunteers. Variability in the glycemic index was substantial, with iAUC for bread ranging from 18 to 182, indicating considerable variability in glycemic responses both within individuals and between individuals (125). The variability of responses to bread and associated issues is detailed in Part 3.2.2.

Genetic and epigenetic factors also contribute to individual variability in responses to FF, particularly genetic variations in enzymes involved in carbohydrate and fat metabolism. In an interventional study, the effects of dairy fat consumption on lipid metabolism were assessed (126). Participants consumed dairy fat daily from full-fat cheese, reduced-fat cheese, butter, butter with calcium caseinate, and a control. They were categorized into “responders” and “non-responders” based on changes in total cholesterol (TC), low-density lipoprotein cholesterol (LDL-c), and high-density lipoprotein cholesterol (HDL-c). “Responders” showed significant reductions in TC and LDL-c, with increases in HDL-c, while “non-responders” had increases in TC and LDL-c and decreases in HDL-c. Baseline lipid levels influenced response: “responders” had higher baseline TC, HDL-c, and lower triglycerides (TAGs), “non-responders”. The study suggests that lipid metabolism responses to dairy fat may depend on baseline characteristics, with higher cholesterol levels being associated with more significant reductions. The food matrix, particularly dairy fat in its natural form, also contributes to cardiovascular health benefits. These findings highlight the complexity of individual variation in dietary responses. Another study evaluated the validity of a global assessment tool for gastrointestinal (GI) wellbeing (127). Participants consumed either a probiotic fermented dairy product (containing *Bifidobacterium lactis, Streptococcus thermophilus, Lactobacillus delbrueckii* subsp. *bulgaricus*, and *Lactococcus lactis*) or a non-fermented dairy product over 6 weeks. Subjects were categorized into “responders” and “non-responders” based on improvements in GI wellbeing (reporting improvements for at least 2 weeks during the 4-week intervention). The study found that “responders” reported significant improvements in digestive symptoms compared to “non-responders.” Sensitivity analysis showed a decrease in digestive symptoms by 41.8% in “responders” and 10.9% in “non-responders.” Stool frequency improvements were no longer significant when a stricter “responder” definition was used. These findings highlight individual differences in response to probiotic interventions and support the validity of the global GI wellbeing assessment tool.

### 3.5 Food characterization is rarely implemented

For an appropriate evaluation of a significant effect of FF (specifically or as a whole) on health, a proper evaluation of FF composition and characteristics is essential. This is even more important when the interest is focused on the variability of response within populations to FF (as some differences in outcomes measured between groups could be expected to be of a lower size) and where FF composition could be one of the factors of variability within or between studies. According to EFSA's scientific and technical guidance for the preparation and submission of an application for a health claim, the evidence provided should demonstrate a sufficiently defined and characterized food/constituent for which the health claim is made (20). In particular, for foods and constituents other than macronutrients, vitamins, and minerals, characterization should include several descriptors, such as the source, characterization of the food matrix and overall composition, physical and chemical properties, microbiological composition, batch-to-batch variability, manufacturing process, and information on stability.

#### 3.5.1 Interventional studies overview of food characterization

Food characterization is critical for identifying possible factors of the FF effects, with an increasing number of studies complementing traditional nutrient profiling by analyzing both microbial strains and metabolites. Among the studies identified in our systematic search (Figure 1), cereal products, primarily bread, were the most studied (*n* = 7), followed by dairy products, mainly yogurt (*n* = 21). Ingredients (macronutrients) of FF were defined in the majority of studies (*n* = 10 in total), while in five studies, the “basic” chemical composition of FF was defined. These studies included low-fat yogurt (53, 54), white and sourdough bread (59), fermented milk (60), and refined rye bread, whole-meal rye bread, and refined wheat bread (61). On the other hand, the technological process by which the FF was produced was described in only three publications, including the production process for rye and wheat bread (28), white and sourdough bread (59), and barley kernel-based bread (62).

In three studies, genera, species, or specific strains were identified. *Lactobacillus bulgaricus* and *Streptococcus thermophilus* were used as starters for milk fermentation (57, 58, 60). Two studies described bread fermentation by yeast and lactobacilli (61) and by yeast alone (62). Metabolites formed during fermentation that may contribute to the effect of FF were described in six studies. Carbohydrates and dietary fiber were identified as active components in three studies (28, 45, 62). Regarding protein components, various amino acids, including leucine and isoleucine (61), were suggested as bioactive metabolites. Ito et al. (60) concluded that components in *S. thermophilus* cells exert antioxidant activity. Finally, SCFAs and bile acids were analyzed as active metabolites in the study by Li et al. (63).

Safety issues were not mentioned in any study, likely because the microorganisms used for fermentation had a Generally Recognized as Safe (GRAS) status. Live microorganisms were present in the final product in approximately half of the studies, including fermented milk (57, 58, 60) and cheese (29).

#### 3.5.2 Gaps in the description of FF in observational studies

Based on our literature search, 27 observational studies were identified. In some studies, only food categories were listed, such as soy products, dairy products, and cereals, while in the majority of the selected studies, specific FF were mentioned, including yogurt, rye bread, cheese, and others. In some studies, variability was only considered at the level of macronutrients. It is important to note that the majority of (large) observational studies used food frequency questionnaires (FFQ), which are more indicative of long-term dietary habits. Other, more precise diet assessments, such as 24-h dietary recalls or food records (which are short-term but more precise in terms of the nature of food eaten and quantities), were less commonly used in large cross-sectional studies. Additionally, the majority of the articles in observational studies grouped these foods into food groups or mentioned, for instance, dairy products without distinguishing whether they were fermented or not or did not report the frequency of consumption of specific foods. Additionally, no composition of raw materials was specified in the description of FF intake, no production process was defined, and there were no reports on the microorganisms (or their viability) in the products. Overall, it is frequently difficult to determine from observational studies whether the food that study participants refer to has been fermented and contains live microorganisms. Within and between countries, there are also variations in the same type of product (e.g., fermented or not, pasteurized, such as butter). The ambiguity becomes even greater when we consider that the study participants may not recognize certain foods as fermented (128).

### 3.6 Concluding remarks and future perspectives

**Figure 2:**
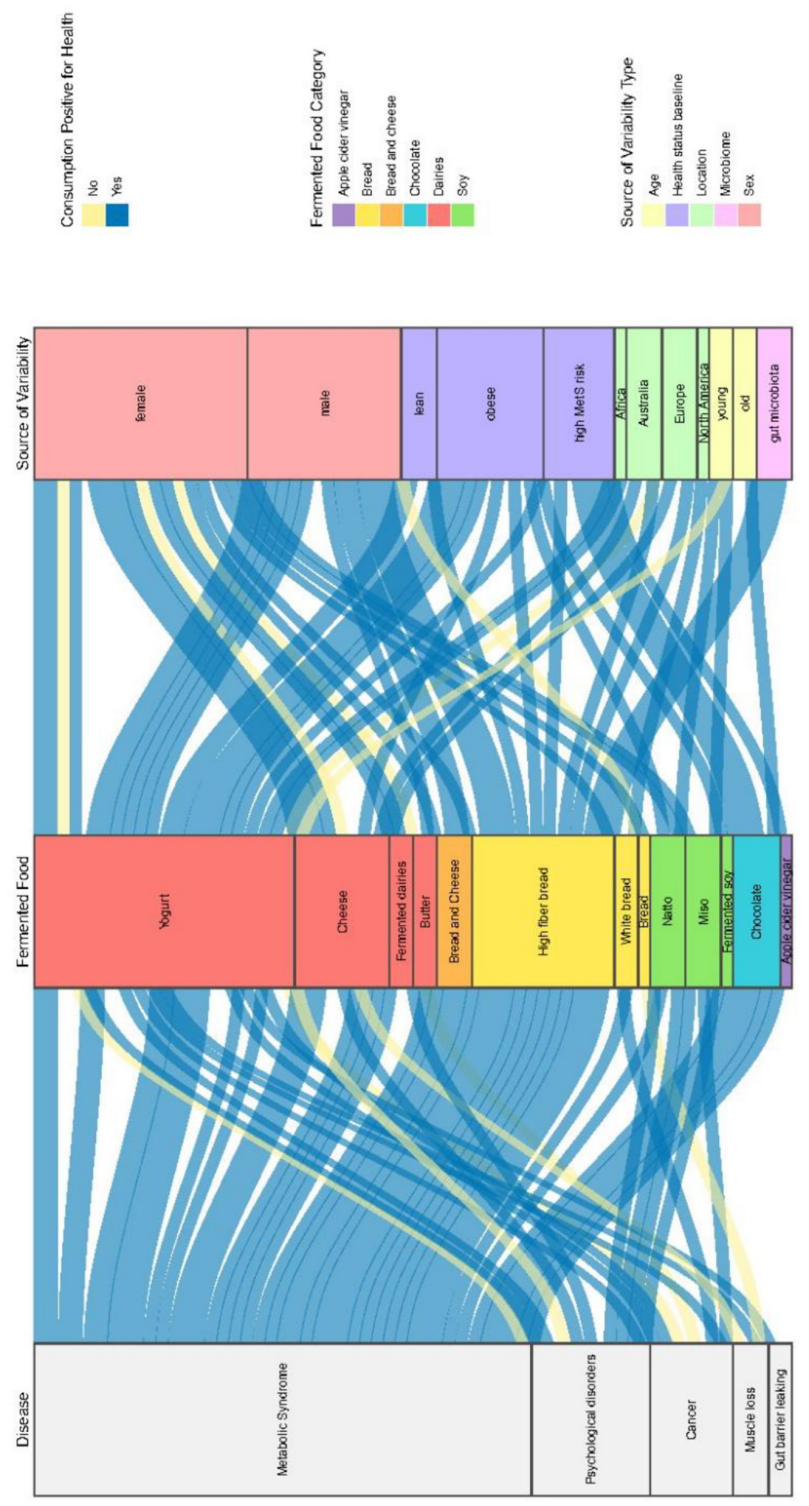
Summary of interactions between health outcomes, fermented foods, and variability sources. A Sankey diagram illustrates the relationships between health outcomes (left axis), fermented food items (middle axis, categorized by type of fermented food), and sources of individual variability (right axis, categorized by type of variability). The width of each flow is proportional to the number of studies included in this review. Flows are colored according to whether the consumption of the corresponding fermented food was positively (blue) or negatively (yellow) associated with health outcomes. Categories of variability sources include sex, location, age, baseline health status, and gut microbiota. Fermented food categories include dairy products, soy products, bread-based products, chocolate, and apple cider vinegar.

**Figure 3:**
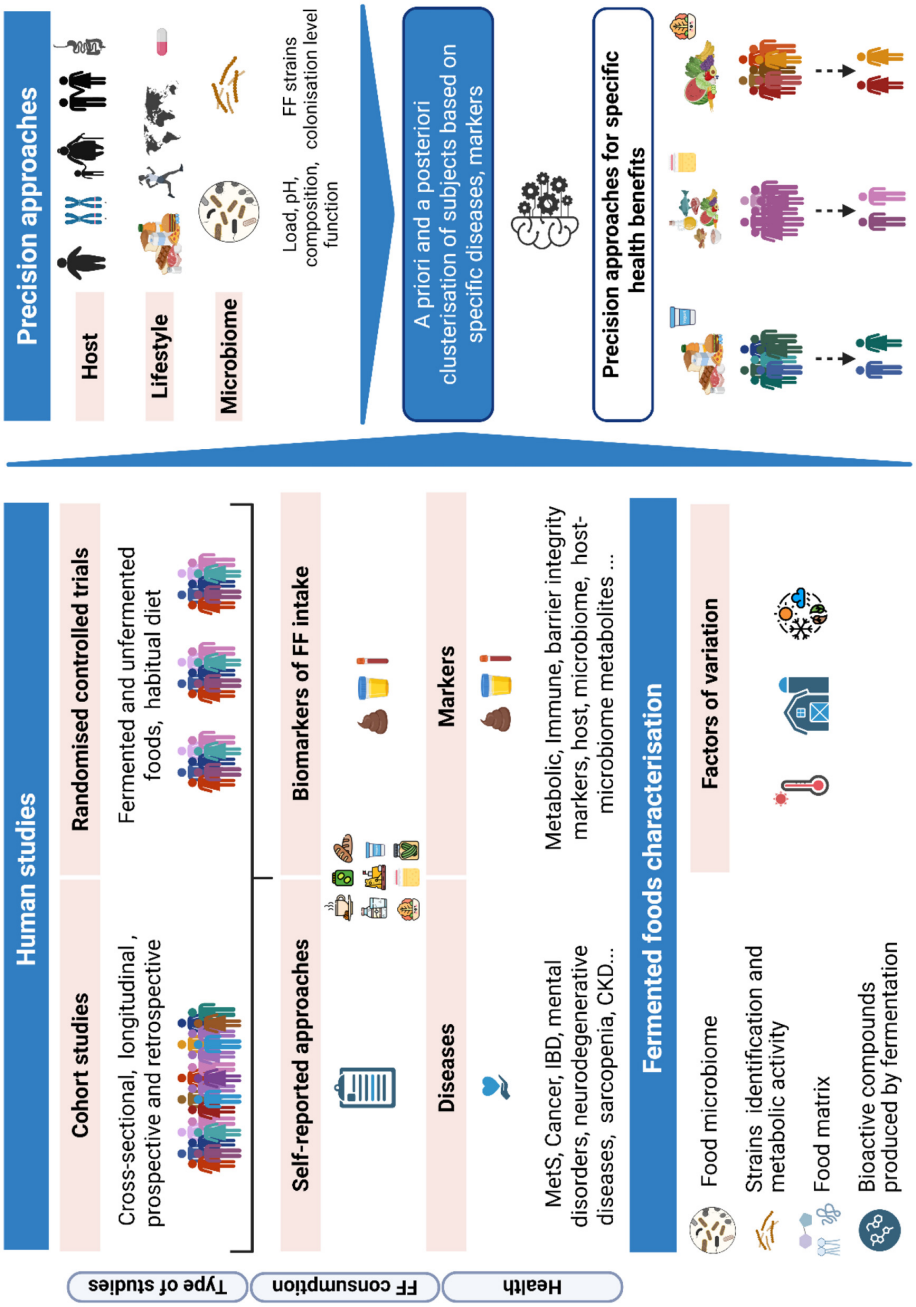
Summary of the parameters that should be carefully measured/mastered in human studies to evaluate more accurately the variability of metabolic/health responses to fermented food intake. The evaluation of these variable responses could form the basis for targeted nutritional recommendations for specific populations, particularly regarding the intake of fermented foods. Created in BioRender. Derrien, M. (2025) https://BioRender.com/99sqyo5, with the addition of resources from Flaticon.com.

The analysis of existing literature in the field of differential response among populations reveals that heterogeneity of response indeed exists (Figure 2). However, the analysis of each factor is hindered by several biases, which account for the inconsistencies observed thus far in the field (Figure 3). First, all the factors of variability within populations can be intertwined (biological sex and living habits or conditions, health status at baseline and biological sex or living conditions, and so on), which renders it difficult to evaluate the respective weight of each factor in the variability observed. Second is the evaluation of the outcome or health effect of the nutritional strategy or diet. Indeed, the differences observed between stratified populations can be relatively small, with the size of the population subgroups even smaller due to stratification. Consequently, if the parameters used to evaluate an alteration in health status are not sufficiently selective or precise, the variability within the stratified populations will overlap with the variability of the measured outcome. Third, the evaluation of FF consumption (type, frequency, and amount) is generally poorly described, particularly in observational studies, where stratification into consumer categories is heterogeneous. This is further complicated when the studies are compared across countries with population-specific consumption patterns. Interventional studies allow the assessment of the effect of specific FF, yet they are often too low in sample size vs. the number of factors of variation, such as dietary habits and microbiome.

Hence, complementary studies are needed (Figure 3). An *a priori* (based on sex, age, and health status at baseline) or *a posteriori* (based on gut microbiome) clustering of the target population is necessary. A more systematic use of biostatistics, bioinformatics, and artificial intelligence methods would help to more precisely define the strategy to use. Indeed, any targeted nutritional recommendation in FF for a specific population (or for each individual) requires a clear, validated, and specific impact of one or several FF on one or several health outcomes in this specific population. Another possibility to address the question is to have an idea of the specific mechanism responsible for (or at least a sensitive marker associated with) the variable response to FF. Knowing this, the first step in the process is to evaluate population variability in response to FF, using appropriate markers of health status (and possibly associated mechanisms), before any targeted recommendation can be implemented. First, larger intervention studies with specific or diverse FF, with their description that would allow identifying factors of variation independent of food intake (using detailed dietary recall or food records), are essential. Second, in population-based cohorts, a specific FFQ dedicated to FF and developed by the PIMENTO initiative could help greatly to address this key point (129). The use of metabolomics methods to identify biomarkers of FF intake would also help to assess the consumption of FF (130). Both intervention and population-based cohort studies would require the description of potential factors that can induce variability in the response to the nutritional strategy (health status, gut microbiota composition, lifestyle, and exposome). Third, FF studies are often limited to dairy products (and bread), geographic location (mainly Europe, North America, and Asia; rarely South America and Africa), and population-specific FF intake (e.g., soy-based fermented products are generally investigated in Asia, whereas dairy products are more studied in Western countries). Expansions toward plant-based FF [other than soy-based, bread, and alcohol-containing (wine and beer)] as well as studies in Africa, the Middle East, and South America are needed. Finally, the characterization of FF is often lacking, except for macronutrient levels, and strain- and metabolite diversity, as well as mechanistic studies, would help to more precisely understand the FF-specific effects on health outcomes. It is only when these limits and requirements are fulfilled that targeted nutritional recommendations or strategies can be implemented for specific individuals and/or groups based on demonstrated concepts of variable response to diets, and more precisely, here, FF.

## Data Availability

The original contributions presented in the study are included in the article/Supplementary material, further inquiries can be directed to the corresponding author.
