# Correction: A scoping review of the health effects of fermented foods in specific human populations and their potential role in precision nutrition: current knowledge and gaps

**DOI:** 10.3389/fnut.2025.1771468

**Published:** 2026-01-16

**Authors:** Christèle Humblot, Panagiota Alvanoudi, Emilia Alves, Ricardo Assunção, Miona Belovic, Tugce Bulmus-Tuccar, Christophe Chassard, Muriel Derrien, Mustafa Fevzi Karagöz, Sibel Karakaya, Marta Laranjo, Fani Th Mantzouridou, Catarina Rosado, Smilja Pracer, Helen Saar, Julien Tap, PrimoŽ Treven, Guy Vergères, Eugenia Pertziger, Isabelle Savary-Auzeloux

**Affiliations:** 1 QualiSud, Université de Montpellier, Avignon Université, CIRAD, Institut Agro, IRD, Université de la Réunion, Montpellier, France; 2 French National Research Institute for Sustainable Development (IRD), Montpellier, France; 3 Laboratory of Food Chemistry and Technology, School of Chemistry, Aristotle University of Thessaloniki, Thessaloniki, Greece; 4 CBIOS - Universidade Lusófona's Research Center for Biosciences & Health Technologies, Lisbon, Portugal; 5 Egas Moniz Center for Interdisciplinary Research (CiiEM), Egas Moniz School of Health & Science, Caparica, Portugal; 6 Food and Nutrition Department, National Institute of Health, Dr. Ricardo Jorge, Lisbon, Portugal; 7 Institute of Food Technology in Novi Sad, University of Novi Sad, Novi Sad, Serbia; 8 Department of Nutrition and Dietetics, Yüksek Ihtisas University, Ankara, Türkiye; 9 Université Clermont Auvergne, INRAE, VetAgroSup, UMRF 0545, Aurillac, France; 10 VIB-Center of Microbiology, KU Leuven, Leuven, Belgium; 11 Nutrition and Dietetics Department, Health Sciences Faculty, Hitit University, Çorum, Türkiye; 12 Department of Food Engineering, Faculty of Engineering, Ege University, Izmir, Türkiye; 13 MED – Mediterranean Institute for Agriculture, Environment and Development & CHANGE – Global Change and Sustainability Institute, Departamento de Medicina Veterinária, Escola de Ciências e Tecnologia, Universidade de Évora, Pólo da Mitra, Évora, Portugal; 14 Institute for Biological Research ‘Siniša Stanković', National Institute of the Republic of Serbia, University of Belgrade, Belgrade, Serbia; 15 TFTAK Center of Food and Fermentation Technologies, Tallinn, Estonia; 16 INRAE, AgroParisTech, UMR1319 MICALIS Institute, Université Paris-Saclay, Jouy-en-Josas, France; 17 Biotechnical Faculty, University of Ljubljana, Ljubljana, Slovenia; 18 Research Division Microbial Food Systems, Agroscope, Bern, Switzerland; 19 Department of Epidemiology and Health Systems, Center for Primary Care and Public Health (Unisanté), University of Lausanne, Lausanne, Switzerland; 20 Université Clermont Auvergne, INRAE, UMR1019 Nutrition Humaine, Saint Genès Champanelle, France

**Keywords:** fermented food, coffee, metabolic syndrome, gut microbiota, population variability, personalized nutrition, yogurt

Author Catarina Rosado was erroneously assigned to affiliation Laboratory of Food Chemistry and Technology, School of Chemistry, Aristotle University of Thessaloniki, Thessaloniki, Greece. The correct affiliation is 4: CBIOS - Universidade Lusófona's Research Center for Biosciences & Health Technologies, Lisbon, Portugal

Author Fani Mantzouridou was erroneously assigned to affiliation French National Research Institute for Sustainable Development (IRD), Montpellier, France. The correct affiliation is 3: Laboratory of Food Chemistry and Technology, School of Chemistry, Aristotle University of Thessaloniki, Thessaloniki, Greece.

The original version of this article has been updated.

